# Use of Health Services by People With Dementia Who Self‐Harm in New South Wales, Australia: A Data Linkage Study

**DOI:** 10.1002/gps.70253

**Published:** 2026-09-02

**Authors:** Adrian R. Walker, Preeyaporn Srasuebkul, Julian N. Trollor, Anne P. F. Wand, Brian Draper, Rachael C. Cvejic, Annette Moxey, Simone Reppermund

**Affiliations:** ^1^ The Department of Developmental Disability Neuropsychiatry Faculty of Medicine and Health UNSW Sydney Sydney New South Wales Australia; ^2^ Centre for Big Data in Health Faculty of Medicine and Health UNSW Sydney Sydney New South Wales Australia; ^3^ Faculty of Medicine and Health National Centre of Excellence in Intellectual Disability Health UNSW Sydney Sydney New South Wales Australia; ^4^ Centre for Healthy Brain Ageing School of Clinical Medicine Faculty of Medicine and Health UNSW Sydney Sydney New South Wales Australia; ^5^ Speciality of Psychiatry Faculty of Medicine and Health University of Sydney Camperdown New South Wales Australia; ^6^ Discipline of Psychiatry and Mental Health Faculty of Medicine and Health UNSW Sydney Sydney New South Wales Australia; ^7^ Older Peoples Mental Health Service Sydney Local Health District C/o Concord Centre for Mental Health Concord New South Wales Australia; ^8^ Eastern Suburbs Older Persons Mental Health Service Prince of Wales Hospital Randwick New South Wales Australia; ^9^ Dementia Australia Griffith Australian Capital Territory Australia

**Keywords:** dementia, linked data, mental health, self‐harm, service use

## Abstract

**Introduction:**

This study aimed to examine the patterns and predictors of health service use for people with dementia who self‐harmed.

**Methods:**

Using a retrospective linked cohort of people who accessed health services in New South Wales, Australia between 2001 and 2015, we identified 154,811 people with dementia or mild cognitive disorder (the dementia cohort), 28,972 people who self‐harmed (the self‐harm cohort), and 1541 who had a record of both dementia or mild cognitive disorder and self‐harm (the dementia and self‐harm subgroup). We examined the rates of health service use (inpatient hospital admissions, emergency department presentations, and mental health ambulatory days) for these cohorts. Using random effects Poisson regression and survival modelling, we investigated the change in and predictors of health service use following self‐harm (for those with dementia) or dementia diagnosis (for those who had self‐harmed).

**Results:**

People with dementia who self‐harmed had the highest rate of use of all health services. Ambulatory mental health service use increased after self‐harm for people in the dementia cohort, while emergency department and mental health related hospital presentations decreased. Hospitalisations for non‐mental health reasons increased after dementia diagnosis for those in the self‐harm cohort, but use of other services decreased. Ambulatory mental health service use predicted increased time to re‐presentation to hospital.

**Discussion:**

Health service use changed after an episode of self‐harm for people with dementia. People with dementia with poor mental health or substance use concerns may benefit from more proactive provision of mental health services.

## Key Points

- People with dementia are at a high risk of self‐harm, but there is limited information on how people with dementia who self‐harm use health services.
- This retrospective cohort study from Australia examined around 185,000 people who had either a history of dementia, self‐harm, or both, and investigated their use of hospital services (inpatient admissions, emergency department presentations, and ambulatory mental health services).
- People with dementia who self‐harmed had the highest rate of use of all services, with increased ambulatory mental health service use predicting increased time to re‐presentation to hospital.
- People with dementia with poor mental health or substance use concerns may benefit from more proactive provision of mental health services.

## 1 Introduction

Dementia is associated with a high number of comorbid health conditions [1]. These comorbidities include stroke and diabetes [1], and preventable conditions like urinary tract infections, pneumonia, and sepsis [2]. People with dementia are also at increased risk of falls and poor nutrition compared to the general population [3, 4, 5]. It is therefore unsurprising that people with dementia tend to use health services at a high rate compared to people without dementia [6, 7].

The Australian Institute for Health and Welfare has targeted self‐harm as an area needing urgent attention, with 145,000 years of healthy life lost in 2019 due to self‐harm and suicide [8]. Men over 85 have one of the highest age‐specific suicide rates in Australia [9]. Furthermore, one driver of hospitalisation for people with dementia is an increased likelihood of hospitalisation due to self‐harm [10, 11]. It is therefore critical that care services are equipped with the tools to prevent self‐harm in older individuals, especially those with dementia.

Currently, there is limited research on how people with dementia who self‐harmed utilise health services. This study aimed to understand how people with dementia who self‐harm utilise health services, including how health service use changes after self‐harm, and whether there is an unmet service need for this cohort. In previous studies, we defined two cohorts of individuals (one of people with dementia or mild cognitive disorder; and one of people who had self‐harmed) from New South Wales (NSW), and examined predictors of death for people in these cohorts that went onto self‐harm (if they had dementia/mild cognitive disorder) or develop dementia (if they had self‐harmed) [12, 13]. This research suggested that engagement with ambulatory mental health services can reduce all‐cause mortality for people with dementia who have self‐harmed. It is critical, therefore, that we understand the factors that affect how people with dementia who have self‐harmed utilise health services. Understanding these factors will allow provision of optimised services for people with dementia who have self‐harmed.

## 2 Materials and Methods

### 2.1 Dementia Advocate Group

Ten lived experience dementia advocates (including people with dementia and care‐partners) were engaged throughout the project. Advocates provided feedback on our analysis plan, and interpretation of our results. All advocates who were able were given the opportunity to read the manuscript prior to submission for publication.

### 2.2 Datasets and Record Linkage, and Study Population

The full description of the datasets, record linkage, and study population can be seen in Supporting Information S1: Supporting 1. Briefly, we used a linked dataset described from Australia described in Reppermund et al. [14] including information on hospital service contacts (inpatient admissions, emergency department presentations, and mental health ambulatory contacts) with births and deaths records.

The cohorts for this study were drawn from the cohorts described in Walker et al. [12] and Walker et al. [13]. Briefly, we formed two cohorts of people aged over 40:

- People who presented to hospital with a diagnosis of dementia or mild cognitive disorder (hereafter combined as ‘dementia’) between 1 July 2001 and 31 December 2014 who had no prior history of self‐harm (the dementia cohort, *n* = 154,811).
- People who presented to hospital for self‐harm between 1 January 2005 and 31 December 2014 who had no prior history of dementia (the self‐harm cohort, *n* = 28,972).

We also defined a subgroup of people (the ‘Dementia and self‐harm subgroup’, full definition in Walker et al. [13]) who had a record of both dementia and self‐harm at any point over the study period.

### 2.3 Predictor Variables

We used all the predictor variables previously defined in Walker et al. [12] and Walker et al. [13]. Time‐fixed variables included:

- Sex;
- Aboriginal or Torres Strait Islander status (control variable only);
- Index of relative socioeconomic disadvantage (IRSD) [15];
- Remoteness of residential address [16];

And time‐varying variables included:

- Age;
- Marital status;
- Number of Elixhauser comorbidities recorded in the previous year [17];
- Number of mental health ambulatory service days in the previous year.
- Number of involuntary mental health admissions to hospital in the previous year.
- Number of emergency department presentations in the previous year.
- A hospital diagnosis of depression, drug and alcohol abuse, psychotic disorders, anxiety, organic disorders, delirium, behavioural problems and personality disorders, with each the presence/absence of each disorder entered as a separate predictor into the model;
- Whether someone had entered the dementia and self‐harm subgroup.

The time‐varying variables (and how yearly periods were determined) were calculated using the method described in Walker et al. [12].

### 2.4 Outcome Variables

We defined three outcome variables:

- The number of recorded episodes of care within hospital for each person. An episode of care (or simply ‘episode’) is defined as the time between an admission to hospital and a separation, noting that a person can be separated directly to another episode of care within the hospital [18]. We split hospital episodes into those for mental health concerns, that is those with a principal diagnosis contained in the ICD10 chapter 5 (Mental and behavioural disorders), and those for physical health issues (principal diagnosis in all other chapters);
- The number of recorded mental health ambulatory service days for each individual. If a person had multiple contacts for different mental health ambulatory services on one day, this was counted as a single day of mental health ambulatory service care;
- The number of recorded emergency department presentations for each individual.

For analysis of those in the dementia and self‐harm subgroup specifically, we also included two more outcome variables:

- Time (in years) from subgroup entry to the first succeeding separation from a hospital admission;
- Time (in years) from subgroup entry to the first succeeding emergency department presentation.

### 2.5 Statistical Analysis

We first compared service use between people who had presented to hospital for both dementia and self‐harm to people who had only presented for either self‐harm or dementia. We then focused only on the service use of people who presented for both dementia and self‐harm (i.e., only those in the dementia and self‐harm subgroup, only after entry into the subgroup). For those in the dementia cohort and self‐harm cohort, follow‐up started from the date of the initial dementia or self‐harm diagnosis or January 1 2005 (whichever occurred last). Follow‐up stopped at the date of death, 31 December 2015, or date of entry to the dementia and self‐harm subgroup, whichever occurred first. For those who entered the dementia and self‐harm subgroup, follow‐up started at the date of the second diagnosis (i.e., the date of first self‐harm for people in the dementia cohort, or the date of the first dementia diagnosis for people in the self‐harm cohort) and ended on 31 December 2015, or date of death, whichever occurred first.

#### 2.5.1 Whole Cohort Analysis

We calculated the rates of service use per 1000 person years for hospital episodes, mental health ambulatory service days, and emergency department presentations, for the dementia cohort, self‐harm cohort, and dementia and self‐harm subgroup. We reported rates of hospital episodes both overall and split by the ICD10 chapter of the principal diagnosis of the episode. As previous work showed evidence that alcohol abuse may be particularly problematic for people with dementia who self‐harmed [12, 13], we also investigated rates of admission for different disorders split using the first three recorded ICD10 digits for hospital episodes with principal diagnosis ICD10 codes in chapter 6 (Nervous system disorders) and chapter 11 (Digestive system disorders). For this analysis, we only report diagnoses where the dementia and self‐harm subgroup had over double the rate of the next highest cohort, and also had greater than 50 individuals with that diagnosis recorded in the dementia and self‐harm subgroup.

**FIGURE 1:**
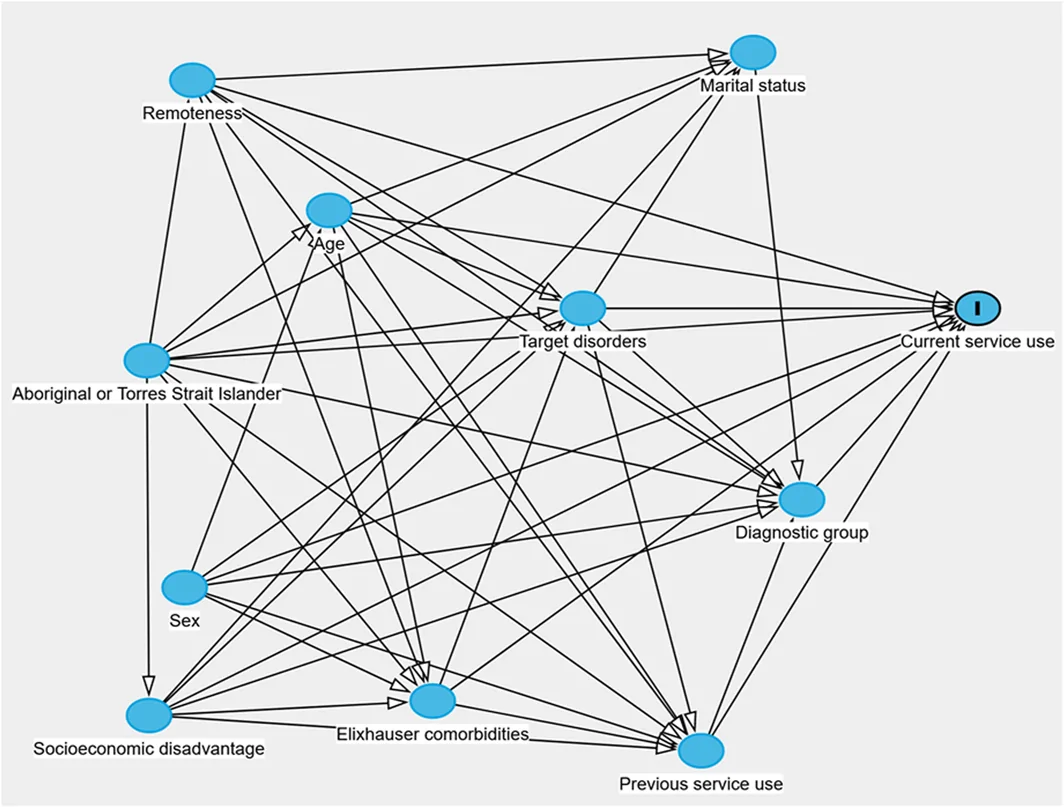
Directed acyclic graph showing proposed causal relationships between our predictor variables and death. Figure made using DAGitty (http://www.dagitty.net/, Textor et al. [39]).

To calculate the differences in overall health service use between those who entered the dementia and self‐harm subgroup and those that did not, we ran two random effects Poisson regression (with a random effect of person) using Stata 17's ‘xtpoisson’ command [19]. We ran one model for those in the dementia cohort, and one model for those in the self‐harm cohort. The variables included in our model of health service use were based on the causal structure outlined in the causal diagram in Figure 1 [20].

#### 2.5.2 Dementia and Self‐Harm Subgroup Analysis

For people who entered the dementia and self‐harm subgroup, we compared the rates of service use per 1000 person years 1 year before entry to the subgroup, 1 year after entry into the subgroup, and after 1 year of entry into the subgroup to observe how service use for this cohort may change over time. Hospital episodes were split into mental (ICD10 chapter 5) and physical (all other chapters) episodes.

To determine predictors of overall health service use for people who entered the dementia and self‐harm subgroup, we ran a series of random effects Poisson regressions (with a random effect of person) including only individuals who entered the dementia and self‐harm subgroup. We individually adjusted for each predictor variable according to Figure 1 [21]. In models specific to only those in the dementia and self‐harm subgroup, people were entered at their date of entry into either the dementia and self‐harm subgroup or January 1 2005 (whichever occurred last).

For the time from entry to the dementia and self‐harm subgroup to the first hospital episode or emergency department presentation, we ran a series of flexible parametric survival analyses with three splines [22], where the outcome was the first service contact to hospital (for either a mental or physical health related episode) or the emergency department after entry in to the model. To account for the fact that predictors of service use may also predict mortality, we included competing risk of death in the model. We individually adjusted for predictor variables according to the proposed causal structure in Figure 1. The time for model started from entry into the dementia and self‐harm cohort.

All reported analyses are exploratory, and the confidence intervals and *p*‐values were not adjusted for multiple comparisons. Analysis was conducted using a combination of R 4.1.2, Stata 17, and SAS 9.4 [19, 23, 24]. For all analyses, we ran a sensitivity analysis excluding mild cognitive disorder (ICD10 code F06.7) as an indicator for dementia (Supporting Information S1: Supporting 2 Tables S1−S6 and Figure S1). We present the analysis including mild cognitive disorder preferentially as it may provide a more accurate estimate of time of symptom onset [25, 26].

## 3 Results

### 3.1 Whole Cohort Analysis

**FIGURE 2:**
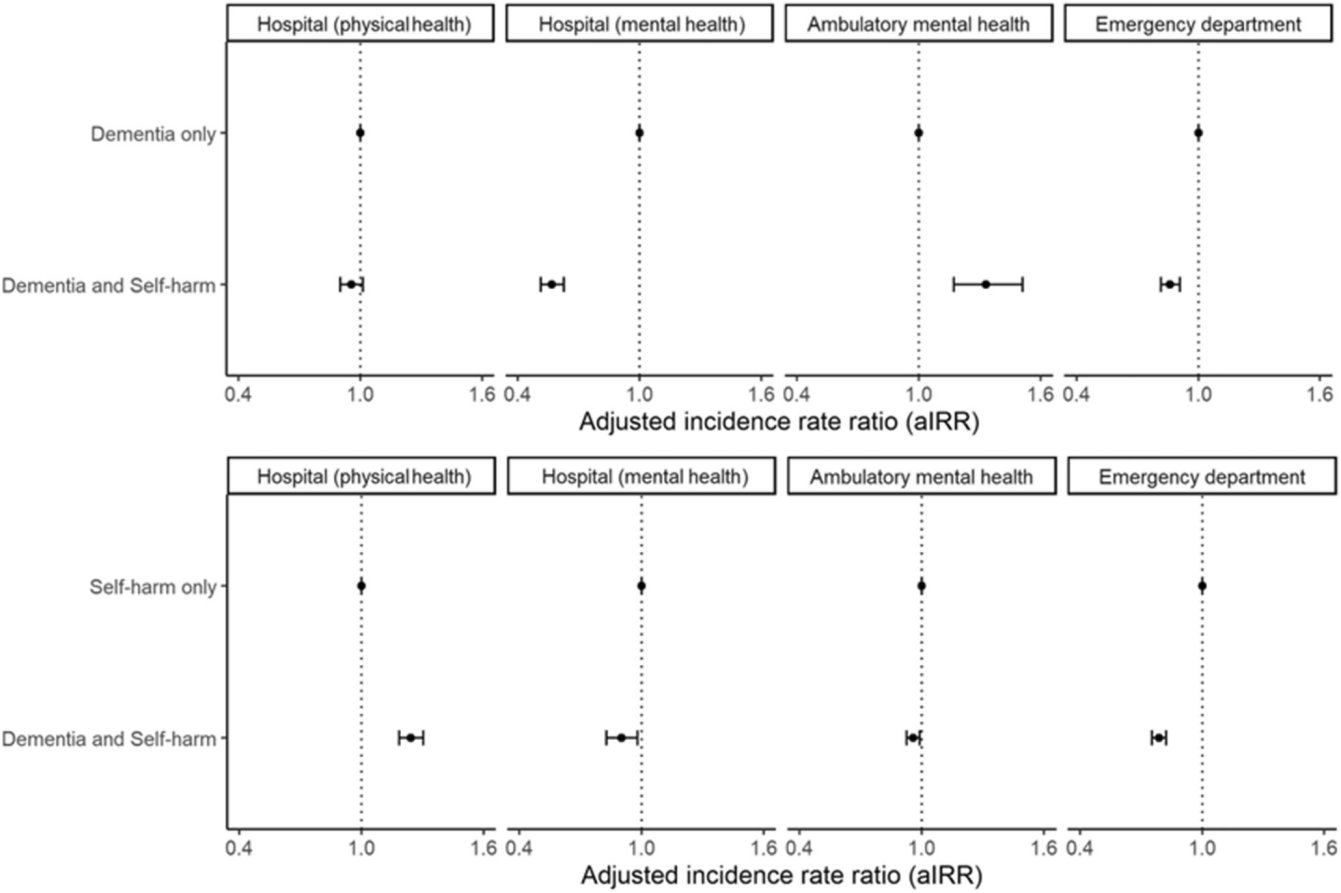
Adjusted incidence rate ratios (aIRRs) for service use for the dementia cohort (top) and self‐harm cohort (bottom), reported separately after entry into the dementia and self‐harm subgroup.

The rates of service use per 1000 person years for the two cohorts and dementia and self‐harm subgroup can be seen in Table 1, and adjusted hazard ratios from our flexible parametric survival analysis can be seen in Figure 2 (full regression results can be seen in Supporting Information S1: Supporting 3 Tables S7 and S8).

**TABLE 1:** Service use in the dementia and self‐harm cohorts.

	Dementia cohort (*n =* 154,811; 484,489 person years)		Self‐harm cohort (*n =* 28,972; 143,487 person years)		Dementia and Self‐harm subgroup^a^ (*n =* 1614; 5272 person years)	
Service‐use type/ICD10 chapter	Rate (per 1000 person years)	Total events	Rate (per 1000 person years)	Total events	Rate (per 1000 person years)	Total events
All hospital episodes	1679.9	815,550	1357.6	194,800	3179.9	16,763
ICD10 chapter						
1. Infectious and parasitic	36.8	17,880	12.4	1781	37.8	199
2. Neoplasms	58.3	28,325	29.3	4207	61.8	326
3. Blood and blood forming organs	19.6	9521	9.3	1334	18.8	99
4. Endocrine, nutritional, and metabolic	37.1	17,995	16.8	2413	48.2	254
5. Mental and behavioural disorders	136.1	66,096	471.3	67,632	682.3	3597
6. Nervous system	58.0	28,146	23.3	3342	160.1	844
G31—Other degenerative diseases of the nervous system, not elsewhere classified.	6.9	3350	0.0	0	17.8	94
G40—Epilepsy	7.8	3762	5.5	787	29.2	154
7. Eye and adnexa	28.9	14,051	13.6	1955	37.9	200
8. Ear and mastoid	1.7	836	1.7	249	< 5.0	^d^
9. Circulatory system	140.5	68,207	43.1	6186	122.4	645
10. Respiratory system	127.9	62,109	40.1	5751	146.4	772
11. Digestive system	86.9	42,180	75.6	10,847	140.0	738
K70—Alcoholic liver disease	3.1	1493	2.9	410	22.6	38
12. Skin and subcutaneous tissue	31.2	15,146	15.9	2283	41.2	217
13. Musculoskeletal and connective tissue	40.7	19,756	43.0	6171	57.3	302
14. Genitourinary system	80.6	39,131	32.1	4608	68.9	363
15. Pregnancy, childbirth, and puerperium	< 1.0	^d^	3.2	453	0.0	0
16. Perinatal conditions	0.0	0	0.0	0	0.0	0
17. Congenital and chromosomal	< 1.0	145	< 1.0	^d^	< 5.0	^d^
18. Abnormal signs and symptoms	130.3	63,260	85.3	12,233	222.5	1173
19. Injury, poisoning, and external causes	203.5	98,787	253.7	36,408	363.3	1915
20. External causes of morbidity	< 1.0	^d^	< 1.0	^d^	0.0	0
21. Factors influencing contact^a^	102.2	49,615	34.1	4890	103.2	544
Dialysis (Z49)^b^	222.1	107,819	111.0	15,933	711.9	3753
Rehabilitation (Z50)^b^	134.4	65,229	39.0	5602	147.4	777
No diagnosis^c^	2.7	1303	3.1	448	6.3	33
Mental health ambulatory days	479.6	232,860	5195.6	602,007	5336.8	29,133
Emergency department presentations	954.8	463,525	1283.8	184,215	2443.1	12,879

Abbreviation: ICD, International classification of diseases.

^a^ Drawn from people in the dementia cohort and self‐harm cohort.

^b^ Dialysis and rehabilitation are presented separately as they account for most chapter 21 codes.

^c^ Combines ICD10 chapter 22 and records without a diagnosis.

^d^ Censored to prevent risk of reidentification.

For rates per 1000 person years, those in the dementia and self‐harm subgroup had the highest rate of use of all three types of health services. Comparing the dementia cohort and the self‐harm cohort, those in the dementia cohort had the higher rate of hospital episodes overall, and for every ICD10 chapter principal diagnosis grouping except mental and behavioural disorders; circulatory system; genitourinary system; pregnancy, childbirth, and the puerperium; and injury, poisoning and certain other consequences of external causes. By contrast, those in the self‐harm cohort had a higher rate of emergency department presentations, and over ten times the rate of mental health ambulatory days.

In our analysis of the dementia cohort, we found that those who entered the dementia and self‐harm subgroup had fewer mental health related hospital episodes (adjusted incident rate ratio (aIRR):0.57; 95% confidence interval (CI):0.51–0.63) and fewer emergency department presentations (aIRR: 0.86; 95% CI: 0.81–0.91), but more mental health ambulatory service days (aIRR: 1.33; 95% CI: 1.17–1.51) after they entered the subgroup. In the analysis of the self‐harm cohort, we found that those who entered the dementia and self‐harm subgroup had more physical health related hospital episodes (aIRR: 1.24; 95% CI: 1.18–1.30), but fewer mental health related hospital episodes (aIRR: 0.90; 95% CI: 0.83–0.98), mental health ambulatory days (aIRR: 0.96; 95% CI: 0.92–0.99), and emergency department presentations (aIRR: 0.79; 95% CI: 0.75–0.82).

### 3.2 Dementia and Self‐Harm Subgroup Analysis

Rates of service use for people in the dementia and self‐harm subgroup, relative to when they entered the combined diagnosis subgroup, can be seen in Table 2. Our modelling of predictors of different types of service use for those in the dementia and self‐harm subgroup can be seen in Table 3. Two variables that consistently predicted all types of service use were the number of Elixhauser comorbidities (excluding those related to mental health), and the number of emergency department presentations in the previous year.

**TABLE 2:** Rates of service use per 1000 person years for the dementia and self‐harm subgroup only, stratified by initial cohort membership and time from sub‐group entry.

Service‐use type	One year prior to subgroup entry		One year after subgroup entry		More than 1 year after subgroup entry	
	Dementia cohort^a^ (402 person years)	Self‐harm cohort (634 person years)	Dementia cohort^a^ (486 person years)	Self‐harm cohort (691 person years)	Dementia cohort^a^ (1526 person years)	Self‐harm cohort (1653 person years)
Hospital admissions (physical health)						
Rate (per 1000 person years)	3061.8	2891.3	3800.7	3840.0	1765.6	2897.1
Total events	1232	1832	1848	2653	2695	4790
Hospital admissions (mental health)						
Rate (per 1000 person years)	1289.9	937.5	1456.1	1308.5	464.5	477.8
Total events	519	594	708	904	709	790
Mental health ambulatory days						
Rate (per 1000 person years)	5343.3	6898.4	7646.6	7247.2	5129.7	4574.3
Total events	2150	4371	3718	5007	7830	7563
Emergency department presentations						
Rate (per 1000 person years)	4774.2	4924.1	3839.7	3365.3	1880.9	2331.0
Total events	1921	3120	1867	2325	2871	3854

^a^ Excludes people who had their first self‐harm diagnosis on the same day as their entry to the dementia cohort, *n* = 206.

**TABLE 3:** Predictors of service use for the dementia and self‐harm cohort.

	Hospital admissions (physical health)			Hospital admissions (mental health)			Mental health ambulatory			Emergency department presentations		
Variable	Incident rate ratio (95% CI)	Standard error	*p* value	Incident rate ratio (95% CI)	Standard error	*p* value	Incident rate ratio (95% CI)	Standard error	*p* value	Incident rate ratio (95% CI)	Standard error	*p* value
Age												
Linear	1.03 (1.00, 1.08)	0.02	0.083	1.02 (0.96, 1.07)	0.03	0.534	1.48 (1.43, 1.53)	0.03	< 0.001	0.96 (0.93, 0.99)	0.02	0.01
Quadratic	1.000 (0.999, 1.000)	0.02	0.021	1.000 (0.999, 1.000)	0.03	0.032	0.996 (0.996, 0.997)	0.025	< 0.001	1.000 (1.000, 1.000)	0.02	0.365
Sex (female)^b^	0.86 (0.75, 0.98)	0.06	0.022	0.91 (0.75, 1.10)	0.09	0.319	1.03 (0.83, 1.27)	0.11	0.799	0.73 (0.64, 0.83)	0.05	< 0.001
Remoteness^b^			0.135^a^			< 0.001^a^			0.009^a^			0.023^a^
Major cities (reference)	1.00	—	—	1.00	—	—	1.00	—	—	1.00	—	—
Inner regional	0.93 (0.79, 1.11)	0.08	0.435	0.61 (0.47, 0.79)	0.08	< 0.001	0.70 (0.53, 0.92)	0.10	0.011	0.85 (0.71, 1.01)	0.07	0.057
Outer regional and beyond	0.75 (0.55, 1.01)	0.11	0.055	1.39 (0.92, 2.11)	0.30	0.122	1.41 (0.88, 2.25)	0.34	0.153	0.71 (0.52, 0.96)	0.11	0.028
Index of relative socioeconomic disadvantage quintile^b^			< 0.001^a^			0.087^a^			0.796^a^			0.249^a^
1–2 (most disadvantaged)	1.00	—	—	1.00	—	—	1.00	—	—	1.00	—	—
3–4	0.70 (0.59, 0.84)	0.06	< 0.001	0.88 (0.68, 1.14)	0.12	0.326	1.05 (0.78, 1.41)	0.16	0.741	1.22 (1.02, 1.46)	0.11	0.03
5–6	0.60 (0.49, 0.73)	0.06	< 0.001	1.00 (0.75, 1.33)	0.15	0.999	0.86 (0.62, 1.19)	0.14	0.367	1.05 (0.86, 1.28)	0.11	0.625
7–8	0.89 (0.71, 1.1)	0.10	0.276	1.23 (0.89, 1.69)	0.20	0.208	0.93 (0.65, 1.33)	0.17	0.694	1.03 (0.82, 1.28)	0.12	0.819
9–10 (least disadvantaged)	0.62 (0.50, 0.76)	0.06	< 0.001	1.28 (0.96, 1.72)	0.19	0.097	1.02 (0.73, 1.42)	0.17	0.912	1.08 (0.88, 1.33)	0.11	0.457
Num. Elixhauser comorbidities in year prior	1.04 (1.02, 1.05)	0.01	< 0.001	1.11 (1.08, 1.14)	0.02	< 0.001	1.03 (1.02, 1.04)	0.01	< 0.001	1.08 (1.07, 1.10)	0.01	< 0.001
MH ambulatory use in year prior (per 10 days)	0.98 (0.96, 1.00)	0.01	0.019	0.97 (0.95, 1.00)	0.01	0.019	1.02 (1.02, 1.02)	< 0.01	< 0.001	1.01 (0.99, 1.02)	0.01	0.309
Involuntary mental health admissions in year prior (per 10 admissions)	1.00 (0.99, 1.01)	< 0.01	0.614	1.02 (1.01, 1.03)	0.01	< 0.001	1.00 (1.00, 1.00)	< 0.01	0.921	1.00 (0.99, 1.01)	< 0.01	0.707
Emergency department presentations in year prior (per 10 presentations)	1.06 (1.02, 1.10)	0.02	0.001	1.10 (1.06, 1.15)	0.02	< 0.001	1.09 (1.06, 1.12)	0.01	< 0.001	1.12 (1.10, 1.14)	0.01	< 0.001
History of depression	0.96 (0.86, 1.08)	0.06	0.504	1.63 (1.38, 1.93)	0.14	< 0.001	1.11 (1.00, 1.23)	0.06	0.042	1.03 (0.93, 1.14)	0.05	0.569
History of drug or alcohol abuse	0.96 (0.85, 1.09)	0.06	0.554	1 (0.80, 1.23)	0.11	0.972	0.61 (0.54, 0.70)	0.04	< 0.001	1.29 (1.14, 1.47)	0.08	< 0.001
History of psychotic disorder	0.72 (0.65, 0.80)	0.04	0.021	1.15 (0.98, 1.35)	0.09	0.954	0.92 (0.83, 1.03)	0.05	< 0.001	0.66 (0.59, 0.73)	0.03	0.009
History of anxiety disorder	1.09 (1.00, 1.19)	0.05	0.061	1.24 (1.05, 1.47)	0.11	0.011	1.58 (1.44, 1.73)	0.07	< 0.001	1.38 (1.25, 1.51)	0.07	< 0.001
History of delirium	1.11 (1.00, 1.23)	0.06	0.042	1.21 (1.04, 1.40)	0.09	0.013	1.05 (0.95, 1.15)	0.05	0.34	0.85 (0.77, 0.93)	0.04	< 0.001
History of behavioural problems	0.93 (0.86, 1.00)	0.04	0.051	1.27 (1.09, 1.48)	0.10	0.002	1.13 (1.04, 1.23)	0.05	0.003	0.92 (0.85, 1.00)	0.04	0.041
History of personality disorders	0.84 (0.74, 0.95)	0.05	0.007	0.89 (0.74, 1.06)	0.08	0.197	0.90 (0.80, 1)	0.05	0.059	1.15 (1.04, 1.27)	0.06	0.006

*Note:* All reported analyses are exploratory, and the confidence intervals and *p*‐values were not adjusted for multiple comparisons.

^a^ Wald test for variable inclusion.

^b^ ‘Unknown’ category excluded from output.

Table 4 shows the results of our model predicting the time to re‐presentation to a hospital episode (for either physical or mental health concerns) or emergency department for individuals in the dementia and self‐harm subgroup after first date of entry to the subgroup.

**TABLE 4:** Predictors of time to re‐presentation to a hospital episode or emergency department for the dementia and self‐harm subgroup after first date of entry to the subgroup.

Variable	Re‐presentation to the emergency department			Re‐presentation to a hospital episode		
	Hazard ratio (95% CI)	Standard error	*p* value	Hazard ratio (95% CI)	Standard error	*p* value
Age						
Linear	0.85 (0.78, 0.92)	0.04	< 0.001	0.88 (0.81, 0.96)	0.04	0.004
Quadratic	1.001 (1.001, 1.002)	< 0.01	< 0.001	1.001 (1.000, 1.001)	< 0.01	0.007
Sex (female)	0.77 (0.55, 1.08)	0.13	0.127	1.32 (0.47, 3.71)	0.70	0.593
Remoteness^b^			0.484^a^			0.705^a^
Major cities (reference)	1.00	—	—	1.00	—	—
Inner regional	0.83 (0.59, 1.16)	0.14	0.28	1.12 (0.79, 1.60)	0.20	0.52
Outer regional and beyond	0.80 (0.47, 1.37)	0.22	0.42	1.21 (0.72, 2.04)	0.32	0.479
Index of relative socioeconomic disadvantage quintile^b^			0.350^a^			0.342^a^
1–2 (most disadvantaged)	1.00	—	—	1.00	—	—
3–4	0.90 (0.65, 1.25)	0.15	0.531	1.25 (0.91, 1.71)	0.20	0.171
5–6	0.98 (0.43, 2.26)	0.42	0.97	0.13 (0.02, 0.76)	0.12	0.023
7–8	0.62 (0.29, 1.32)	0.24	0.217	0.77 (0.32, 1.83)	0.34	0.552
9–10 (least disadvantaged)	0.89 (0.63, 1.26)	0.16	0.504	1.07 (0.71, 1.61)	0.22	0.759
EH comorbidities in year prior	1.29 (1.22, 1.35)	0.03	< 0.001	1.29 (1.22, 1.35)	0.03	< 0.001
MH ambulatory use in year prior (per 10 days)	0.90 (0.78, 1.03)	0.06	0.137	0.84 (0.78, 0.91)	0.03	< 0.001
Involuntary MH admissions in year prior (per 10 admissions)	0.95 (0.89, 1.02)	0.03	0.145	1.00 (0.97, 1.03)	0.01	0.964
ED presentations in year prior (per 10 presentations)	1.05 (1.04, 1.07)	0.01	< 0.001	1.05 (1.03, 1.06)	0.01	< 0.001
History of depression	1.00 (0.77, 1.30)	0.13	0.997	1.20 (0.92, 1.56)	0.16	0.17
History of drug or alcohol abuse	1.32 (1.00, 1.74)	0.19	0.047	1.27 (0.98, 1.66)	0.17	0.075
History of psychotic disorder	1.00 (0.79, 1.27)	0.12	0.987	1.13 (0.87, 1.47)	0.15	0.348
History of anxiety disorder	1.01 (0.79, 1.28)	0.12	0.966	0.76 (0.60, 0.97)	0.09	0.028
History of delirium	1.47 (1.11, 1.94)	0.21	0.006	1.23 (0.91, 1.65)	0.19	0.184
History of behavioural problems	1.25 (0.99, 1.58)	0.15	0.065	1.17 (0.92, 1.49)	0.14	0.203
History of personality disorders	0.99 (0.72, 1.37)	0.16	0.968	0.83 (0.61, 1.14)	0.13	0.247

^a^ Wald test for variable inclusion.

^b^ ‘Unknown’ category excluded from output.

### 3.3 Sensitivity Analysis

Sensitivity analyses excluding mild cognitive impairment as an indicator of dementia showed that the change on mental health ambulatory use after dementia diagnosis for the self‐harm cohort was no longer significant. Most predictors of service use remained unchanged. Predictors of time to service use remained unchanged, noting that a history of drug or alcohol abuse no longer predicted time to ED presentation, but predicted time to hospital admission.

## 4 Discussion

### 4.1 Summary of Findings

We found that people with dementia who self‐harmed had the highest rate of hospital episodes and emergency department admissions, and mental health ambulatory days compared to people who presented for either dementia or self‐harm alone. Furthermore, people with dementia who self‐harmed presented to hospital at a substantially greater rate for epilepsy, alcoholic liver disease, and other degenerative disorders of the nervous system (not elsewhere classified) than people who presented for dementia or self‐harm alone.

When adjusting for potential confounding variables, we observed an association with a decrease in emergency department presentations and hospital episodes related for mental health disorders for those with dementia after they self‐harmed, along with an association with an increase in mental health ambulatory service days. For people who had self‐harmed first, we observed an association with an increase in hospital episodes for physical health concerns, but an association with a decrease in hospital episodes for mental health concerns, mental health ambulatory service days, and emergency department presentations after their first presentation for dementia. Rates of service use directly after entry to the subgroup compared to 1 year after entry to the subgroup indicate that this shift may be temporary, with rates of all types of health service use dropping substantially after 1 year.

Physical comorbidities (Elixhauser) were associated with all types of service use, but mental health comorbidities were mainly associated with mental health service use (noting this was less strongly observed when not including mild cognitive impairment as an indicator of dementia). Older age was associated with using mental health ambulatory services more but associated with decreased use of emergency departments. Female sex was associated with having fewer physical health hospital admissions and emergency department presentations. Living in inner regional areas was associated with less all health services utilisation compared to living in major cities, and living in less socioeconomically disadvantaged areas was generally associated with fewer presentations to hospital for physical health concerns.

Older age was associated with a longer time to present to both the emergency department and hospital following entry to the dementia and self‐harm subgroup. A greater number of physical comorbidities were associated with quicker re‐presentation to both services, as was a history of delirium. A higher number of previous emergency department admissions was associated with a shorter time to re‐presentation to both services, while mental health ambulatory use was associated with a longer time to being admitted to hospital (but not the emergency department).

### 4.2 Interpretation

Our results suggest that there is a shift in health service use when an individual with dementia self‐harms, or an individual who has self‐harmed is diagnosed with dementia in hospital. This change is to be expected—as an individual's health profile changes, their engagement with health services should alter. Indeed, it is reassuring to see that those with dementia are provided more ambulatory mental services following self‐harm, and that they have fewer hospital episodes for mental health concerns. This finding is particularly encouraging in the context of our previous research on this population which indicated that an increased number of mental health ambulatory service days was associated with a decreased likelihood of mortality for people with dementia who self‐harmed [13].

The flip side of this finding is that people with dementia may not always be provided appropriate post‐dementia diagnosis support, and it is often only following a health crisis (like self‐harm) that they are connected to appropriate services [27]. In Australia, post‐diagnosis support for people with dementia and mild cognitive disorder is generally coordinated by the medical practitioner making the diagnosis. Though referral to mental health and counselling support can be made by the diagnosing practitioner, these supports are not routine when providing post‐diagnosis support for people with dementia. Previous work has highlighted the need for appropriate and sensitive counselling in post‐diagnosis support, particularly following a diagnosis of young onset dementia [28, 29]. Our findings provide impetus for targeted provision of mental health and counselling support immediately post‐diagnosis for people diagnosed with dementia or mild cognitive disorder with identified needs. Furthermore, for people with existing mental health concerns prior to their dementia or mild cognitive disorder diagnosis, referral to specialist mental health services for formal mental health assessment may be indicated as part of targeted prevention of self‐harm.

We observed a small decrease in the number of mental health ambulatory service days provided to individuals who self‐harmed after their initial presentation for dementia. This change was marked by a shift away from hospitalisations related to mental health, to hospitalisations for physical health concerns. This shift likely reflects the fact that specialised dementia care in Australia is generally provided through geriatric services for complex cases (such as those with comorbid disorders). It is possible that mental health supports are continued within these services as part of dementia care where required. As above, we recommend that, given that a historical diagnosis of self‐harm may indicate a history of poor mental health, clinicians should assess current mental health following dementia diagnoses in this population. If mental health concerns are identified, clinicians should address these or refer the person to mental health services as part of post‐diagnostic support.

It is worth highlighting that use of health services appears to drop following 1 year of entry to the dementia and self‐harm subgroup. It is unclear if these services are unnecessary (as health is being better managed), or whether service provision reduces in the absence of acute health events. It may be the case that service use drops off as individuals are referred to residential aged care, with a cursory examination of our data indicating that aged care referrals may spike upon entry to the dementia and self‐harm subgroup (Supporting Information S1: Supporting 4).

For specific health conditions, we note people with dementia who self‐harmed had a high relative rate of admission for non‐specific neurological disorders, epilepsy and alcoholic liver disorder. Previous research has shown that both neurological disorders (including epilepsy) and alcohol use disorder are correlated with self‐harm and suicide [30, 31, 32, 33]. However, we did not observe a higher rate of presentations for these disorders in the self‐harm cohort. Our previous research on this cohort showed that dementia related to alcohol use (ICD10 code G31.2) and neurological disorders more broadly (ICD10 codes G31, excluding G31.2) are more prevalent in those with dementia that go onto self‐harm than those that do not [12]. As such, we posit that there may be a complex interaction between neurological disorders (in particular those related to alcohol use), dementia, and self‐harm that we cannot untangle with our data. Further, the role of frailty in these relationships is unknown but may be relevant given bidirectional causality between depression and frailty in older adults [34] and an association between frailty and suicidal behaviours [35], even in the absence of depression [36]. Investigation of these various interactions may prove useful in preventing self‐harm in people with dementia.

### 4.3 Strengths and Limitations

Our study was able to leverage a large amount of state‐wide public health data to examine the health service use of people with dementia who self‐harmed. Despite this, there were instances where small numbers of individuals may have impacted our findings. For example, though the rate of dialysis use seems inflated for the dementia and self‐harm subgroup, this high rate was driven by fewer than five individuals who regularly received dialysis. We also note that our data only captures health service use, and not primary or private specialist health service use. There is evidence that different dementia subgroups may be associated with different profiles of psychological symptoms [37]. However, accurate identification of dementia subtype from administrative data has been shown to be difficult without clinical indicators [38]. As such, we chose not to stratify by subtype in these analyses. Finally, as analyses were exploratory and we chose not to adjust for multiple comparisons, we cannot rule out the role of chance in the detection of significant effects.

## 5 Conclusion

Our study aimed to examine the health service use patterns of people with dementia who self‐harmed. We showed that an episode of self‐harm for someone with dementia is associated with a marked shift in health service use, also found for those who had self‐harmed prior to developing dementia. This switch in health service use is consistent with providing appropriate health supports for this population. However, the data suggests that many of these supports are not provided proactively after an initial dementia or self‐harm diagnosis and tail off after 1 year of entry into the dementia and self‐harm subgroup. More needs to be done to engage those people with dementia who need mental health support with mental health services before they go onto self‐harm, and likewise for people who have self‐harmed who may go onto develop dementia.

## Author Contributions

All authors contributed to conceiving, designing, and interpreting results from the study, drafting or review of the manuscript. A.R.W. planned and conducted the statistical analysis for the paper, and wrote the initial draft.

## Funding

This project was funded by the Hazel Hawke Research Grant in Dementia Care through the Dementia Australia Research Foundation (project ‘Self‐harm in people with dementia—using big data to improve outcomes and inform strategies to prevent self‐harm and suicide’).

## Consent

The study was approved by the NSW Population and Health Services Research Ethics Committee (HREC/13/CIPHS/7; Cancer Institute NSW reference: 2013/02/446 and Sub‐study Reference number: 2019/ETH01806). This approval included a waiver of informed consent, and thus individual consent was not necessary to obtain.

## Conflicts of Interest

Adrian R. Walker is currently employed at the institution where the manuscript originates, has had their salary partly paid by the research grant from which this project originates, has received thanks for presenting this project to Carers NSW, and has had conference attendance sponsored through the UNSW Sydney Scientia fund for this project. Julian N. Trollor has received support for the manuscript from the National Health and Medical Research Council (NHMRC) grants GNT2009771, GNT2006240, & GNT 1123033. Anne Wand was funded by Roche (domestic flights and one night accommodation) to attend the ‘Preceptorship on Mild Cognitive Impairment (MCI) and Mild Dementia due to Alzheimer’s Disease’ meeting on 24‐25 February 2023. Rachael C. Cvejic was supported by a Dementia Australia Research Foundation Post‐doctoral Fellowship funded by the Dementia Australia Research Foundation and Bartle Pathway to Care. Annette Moxey is currently employed by Dementia Australia. Simone Reppermund received funding through the Dementia Australia Hazel Hawke Research Grant in Dementia Care. For the remaining authors, no conflicts of interest were declared.

## Supporting information


Supporting Information S1

## Data Availability

The data that support the findings of this study are available on request from the corresponding author. The data are not publicly available due to privacy or ethical restrictions.
