# Supplementary material for: Use of Health Services by People With Dementia Who Self‐Harm in New South Wales, Australia: A Data Linkage Study
Source: Int J Geriatr Psychiatry. 2026 Sep 2;41(9):e70253. doi: 10.1002/gps.70253 (PMC13536255; doi:10.1002/gps.70253)
Supplement: Supplementary file 1 [file GPS-41-e70253-s001.docx]

SUPPLEMENTARY - **Use of health services by people with dementia who self-harm in New South Wales, Australia: a data linkage study**

# 1 Supplementary methods

## 1.1 Datasets and record linkage

The linked datasets used in this study are described in Reppermund et al. (2019) Specifically, we used the following datasets:

- the Admitted Patient Data Collection (APDC) containing information on all hospital admissions in NSW between 1 July 2001-31 December 2015;
- the Emergency Department Data Collection (EDDC) containing information on most public emergency department presentations in NSW between 1 January 2005-31 December 2015;
- the Mental Health Ambulatory Data Collection (MH-AMB) containing information on all activities in NSW public ambulatory mental health services between 1 January 2001-31 December 2015;
- the NSW Registry of Births, Deaths, and Marriages (RBDM) containing information of date of death in NSW between 1 January 1994-31 December 2015.
- the NSW Cause of Death Unit Record File (COD-URF) containing information all causes of death in NSW between 1 January 1985-31 December 2013.

Deidentified linkage of the datasets was performed by the NSW Centre for Health Record Linkage based on a statistical linkage key (SLK581) and unit record data were sent to researchers after removal of personal identifiers.

## 1.2 Study population

### 1.1.1 Primary cohorts

The cohorts for this study were drawn from the cohorts described in Walker et al. (2023) and Walker et al. (2024). Briefly, we formed two cohorts of people aged over 40:

- People who presented to hospital with a diagnosis of dementia or mild cognitive disorder (hereafter combined as “dementia”) between 1 July 2001 and 31 December 2014 who had no prior history of self-harm (the dementia cohort, *n* = 154,811).
- People who presented to hospital for self-harm between 1 January 2005 and 31 December 2014 who had no prior history of dementia (the self-harm cohort, *n* = 28,972).

The definitions for dementia and self-harm were based on the criteria outlined by Walker et al. (2023). In summary, cases of dementia were identified using International Classification of Diseases, 10th Revision (ICD-10) codes from inpatient hospital admissions (APDC) and outpatient mental health interactions (MH-AMB). These codes encompassed A81.0, F00-F03, F06.7, G30, G31, and F05.1. Self-harm was determined through the presence of specific ICD-10 codes (X60-X84 and Y87.0), ICD-9 codes (E950-E959), or Systematized Nomenclature of Medicine (SNOMED) codes, as described by Chitty et al. (2022). Records from inpatient hospital admissions (APDC), outpatient mental health interactions (MH-AMB), and emergency department presentations (EDDC) were used to identify these codes. ICD-10 codes capture all intentional self-harm, while ICD-9 codes include self-inflicted injuries regardless of intent. SNOMED codes were selected to identify explicit indications of self-inflicted injury, poisoning, or suicidal behaviour, following the methods of Chitty et al. (2022). People with first presentations for both dementia and self-harm on the same date were classified into the dementia cohort, under the assumption that dementia symptoms were likely present prior to that initial presentation.

### 1.1.2 Dementia and self-harm subgroup

We defined a subgroup of people (the “Dementia and self-harm subgroup”, full definition in Walker et al. (2024)) who had a record of both dementia and self-harm at any point over the study period. This subgroup consisted of people who either:

- were identified as having dementia according to hospital records and later presented to hospital for self-harm (*n=*569*)*;
- presented to hospital for self-harm and later received were identified as having dementia according to hospital records (*n=*839*)*;
- presented to hospital with a record of self-harm and dementia on the same day (*n*=206);

between 1 January 2005 and 31 December 2015. Entry to this subgroup occurred on the date of the second diagnosis (e.g., if a person’s initial diagnosis was dementia, then the date of their second diagnosis was the date when self-harm was first recorded), or on the date of study entry for people who initially presented with both dementia and self-harm. We excluded those who died on the same day they entered the cohort to ensure we had sufficient follow-up time (n = 73; 42 from the dementia cohort). Overall, there were 1541 people who entered this subgroup.

**References**

CHITTY, K. M., CVEJIC, R. C., HEINTZE, T., SRASUEBKUL, P., MORLEY, K., DAWSON, A., CARTER, G., DINH, M., BUCKLEY, N. A. & TROLLOR, J. N. 2022. The Association Between Problematic Use of Alcohol and Drugs and Repeat Self-Harm and Suicidal Ideation. *Crisis,* 44**,** 309-317.

REPPERMUND, S., HEINTZE, T., SRASUEBKUL, P., REEVE, R., DEAN, K., SMITH, M., EMERSON, E., SNOYMAN, P., BALDRY, E., DOWSE, L., SZANTO, T., SARA, G., FLORIO, T., JOHNSON, A., CLEMENTS, M., MCKENZIE, K. & TROLLOR, J. 2019. Health and wellbeing of people with intellectual disability in New South Wales, Australia: a data linkage cohort. *BMJ Open,* 9**,** e031624.

WALKER, A. R., SRASUEBKUL, P., TROLLOR, J. N., WAND, A. P., DRAPER, B., CVEJIC, R. C., MOXEY, A. & REPPERMUND, S. 2023. Risk factors for dementia and self‐harm: A linkage study. *Alzheimer's & Dementia*.

WALKER, A. R., SRASUEBKUL, P., TROLLOR, J. N., WAND, A. P., DRAPER, B., CVEJIC, R. C., MOXEY, A. & REPPERMUND, S. 2024. Mortality in people living with dementia who self-harmed: An Australian data linkage study. *Australian & New Zealand Journal of Psychiatry,* 58**,** 990-1000.

# 2 Sensitivity analysis excluding mild cognitive impairment as an indicator of dementia

## 2.1 All people

**Table S1: Service use in the Dementia and Self-harm cohorts (without mild cognitive impairment as an indicator of dementia). Number of events not included to prevent risk of reidentification.**

|  | **Dementia cohort**  **(*n =* 151,608)** | **Self-harm cohort**  **(*n =* 29,033)** | **Dementia and  Self-harm subgroup**  **(*n* = 1550)** |
| --- | --- | --- | --- |
| **Service-use type/ICD10 chapter** | **Rate (per 1000 person years)** | **Rate (per 1000 person years)** | **Rate (per 1000 person years)** |
| **Hospital episodes** | **1665.4** | **1368.3** | **3041.0** |
| 1. Infectious and Parasitic | 37.1 | 12.5 | 37.7 |
| 2. Neoplasms | 57.8 | 29.4 | 63.4 |
| 3. Blood and Blood forming organs | 19.7 | 9.3 | 19.6 |
| 4. Endocrine, nutritional, and metabolic | 37.1 | 17.0 | 45.0 |
| 5. Mental and Behavioural | 135.9 | 474.1 | 621.9 |
| 6. Nervous System | 58.6 | 23.4 | 169.3 |
| G31 – Other degenerative diseases of the nervous system, not elsewhere classified. | 7.2 | 0.0 | 19.6 |
| G40 – Epilepsy | 7.6 | 5.6 | 28.6 |
| 7. Eye and Adnexa | 27.9 | 13.6 | 40.0 |
| 8. Ear and Mastoid | 1.7 | 1.7 | 2.1 |
| 9. Circulatory system | 140.0 | 43.4 | 121.6 |
| 10. Respiratory system | 129.4 | 40.5 | 144.9 |
| 11. Digestive system | 87.1 | 75.6 | 144.9 |
| K70 – Alcoholic liver disease | 3.1 | 2.9 | 24.2 |
| 12. Skin and subcutaneous tissue | 31.1 | 16.0 | 41.1 |
| 13. Musculoskeletal and connective tissue | 40.2 | 43.1 | 56.5 |
| 14. Genitourinary system | 81.5 | 32.3 | 65.7 |
| 15. Pregnancy, childbirth, and puerperium | <1 | 3.1 | 0.0 |
| 16. Perinatal conditions | <1 | 0.0 | 0.0 |
| 17. Congenital and chromosomal | 130.9 | <1 | <1 |
| 18. Abnormal signs and symptoms | 206.1 | 85.8 | 219.8 |
| 19. Injury, poisoning, and external causes | <1 | 253.9 | 369.5 |
| 20. External causes of morbidity | 102.9 | <1 | 0.0 |
| 21. Factors influencing contact* | <1 | 34.3 | 102.6 |
| Dialysis (Z49)* | 215.0 | 116.2 | 617.1 |
| Rehabilitation (Z50)* | 122.5 | 39.2 | 152.4 |
| No diagnosis** | 2.7 | 3.2 | 5.2 |
|  |  |  |  |
| **Mental health ambulatory days** | 567.3 | 4274.2 | 6097.7 |
|  |  |  |  |
| **Emergency department presentations** | 955.9 | 1291.8 | 2320.5 |
| ICD: International classification of diseases |  |  |  |
| *Dialysis and rehabilitation are presented separately as they account for most chapter 21 codes | | | |
| **Combines ICD10 chapter 22 and records without a diagnosis | |  |  |

## Figure S1: Adjusted incidence rate ratio's (aIRRs) for service use for the dementia cohort (top) and self-harm cohort (bottom), reported separately after entry into the dementia and self-harm subgroup (without mild cognitive impairment as an indicator of dementia)

## Table S2: Adjusted incidence rate ratio's (aIRRs) for service use for the dementia cohort, full regression results, reported separately after entry into the dementia and self-harm subgroup (without mild cognitive impairment as an indicator of dementia)

|  | **Hospital Admissions (Physical health)** | | | **Hospital Admissions (Mental health)** | | | **Mental Health Ambulatory** | | | | **Emergency Department Presentations** | | | |
| --- | --- | --- | --- | --- | --- | --- | --- | --- | --- | --- | --- | --- | --- | --- |
| **Variable** | **Incident Rate Ratio (95% CI)** | **Standard Error** | **p value** | **Incident Rate Ratio (95% CI)** | **Standard Error** | **p value** | **Incident Rate Ratio (95% CI)** | | **Standard Error** | **p value** | **Incident Rate Ratio (95% CI)** | **Standard Error** | **p value** | |
| **Cohort membership** |  |  |  |  |  |  |  | |  |  |  |  |  | |
| Dementia only | 1.00 | … | … | 1.00 | … | … | 1.00 | | … | … | 1.00 | … | … | |
| Dementia and self-harm | 0.96 (0.9, 1.02) | 0.03 | 0.155 | 0.56 (0.51, 0.62) | 0.03 | <0.001 | 1.35 (1.29, 1.4) | | 0.03 | <0.001 | 0.86 (0.81, 0.91) | 0.02 | <0.001 | |
|  |  |  |  |  |  |  |  | |  |  |  |  |  | |
| **Age** |  |  |  |  |  |  |  | |  |  |  |  |  | |
| Linear | 1.17 (1.17, 1.18) | <0.01 | <0.001 | 1.02 (1.00, 1.03) | 0.01 | 0.025 | 1.21 (1.20, 1.22) | | 0.01 | <0.001 | 1.01 (1.00, 1.01) | <0.01 | 0.11 | |
| Quadratic | 0.999 (0.999, 0.999) | <0.01 | <0.001 | 1.000 (1.000, 1.000) | <0.01 | <0.001 | 0.998 (0.998, 0.998) | | <0.01 | <0.001 | 1.000 (1.000, 1.000) | <0.01 | 0.028 | |
|  |  |  |  |  |  |  |  | |  |  |  |  |  | |
| **Sex (Female)**^✝^ | 0.72 (0.70, 0.73) | 0.01 | <0.001 | 0.66 (0.64, 0.68) | 0.01 | <0.001 | 1.06 (1.00, 1.12) | | 0.03 | 0.046 | 0.71 (0.70, 0.72) | 0.01 | <0.001 | |
|  |  |  |  |  |  |  |  | |  |  |  |  |  | |
| **Remoteness**^✝^ |  |  | <0.001* |  |  | <0.303* |  | |  | <0.001* |  |  | <0.001* | |
| Major cities (Reference) | 1 | … | … | 1 | … | … | 1 | | … | … | 1 | … | … | |
| Inner Regional | 0.89 (0.88, 0.91) | 0.01 | <0.001 | 1.00 (0.95, 1.04) | 0.02 | 0.923 | 1.67 (1.55, 1.80) | | 0.06 | <0.001 | 0.79 (0.78, 0.81) | 0.01 | <0.001 | |
| Outer regional and beyond | 0.93 (0.90, 0.96) | 0.01 | <0.001 | 1.05 (0.98, 1.13) | 0.04 | 0.137 | 1.34 (1.20, 1.51) | | 0.08 | <0.001 | 0.68 (0.66, 0.70) | 0.01 | <0.001 | |
|  |  |  |  |  |  |  |  | |  |  |  |  |  | |
| **Index of relative socioeconomic disadvantage quintile**^✝^ |  |  | <0.001* |  |  | <0.001* |  | |  | <0.001* |  |  | <0.001* | |
| 1-2 (Most disadvantaged) | 1.00 | … | … | 1.00 | … | … | 1.00 | | … | … | 1.00 | … | … | |
| 3-4 | 0.91 (0.89, 0.93) | 0.01 | <0.001 | 0.89 (0.85, 0.93) | 0.02 | <0.001 | 1.18 (1.09, 1.28) | | 0.05 | <0.001 | 0.89 (0.88, 0.91) | 0.01 | <0.001 | |
| 5-6 | 0.92 (0.90, 0.94) | 0.01 | <0.001 | 0.92 (0.87, 0.97) | 0.02 | 0.001 | 1.08 (0.99, 1.18) | | 0.05 | 0.071 | 0.87 (0.86, 0.89) | 0.01 | <0.001 | |
| 7-8 | 0.93 (0.90, 0.95) | 0.01 | <0.001 | 1.07 (1.01, 1.13) | 0.03 | 0.024 | 1.91 (1.74, 2.11) | | 0.1 | <0.001 | 0.85 (0.83, 0.87) | 0.01 | <0.001 | |
| 9-10 (Least disadvantaged) | 0.88 (0.86, 0.90) | 0.01 | <0.001 | 0.87 (0.83, 0.92) | 0.02 | <0.001 | 1.63 (1.49, 1.78) | | 0.07 | <0.001 | 0.70 (0.68, 0.72) | 0.01 | <0.001 | |
|  |  |  |  |  |  |  |  | |  |  |  |  |  | |
| **Num. Elixhauser comorbidities in year prior** | 1.00 (1.00, 1.00) | <0.01 | 0.395 | 0.95 (0.94, 0.96) | <0.01 | <0.001 | 0.98 (0.98, 0.98) | | <0.01 | <0.001 | 1.04 (1.03, 1.04) | <0.01 | <0.001 | |
|  |  |  |  |  |  |  |  | |  |  |  |  |  | |
| **MH Ambulatory use in year prior  (per 10 days)** | 0.997 (0.992, 1.002) | <0.01 | 0.279 | 1 (0.993, 1.008) | <0.01 | 0.924 | 1.041 (1.04, 1.042) | | <0.01 | <0.001 | 0.999 (0.994, 1.005) | <0.01 | 0.738 | |
|  |  |  |  |  |  |  |  | |  |  |  |  |  | |
| **Involuntary mental health admissions in year prior (per 10 admissions)** | 0.992 (0.989, 0.995) | <0.01 | <0.001 | 0.993 (0.990, 0.995) | <0.01 | <0.001 | 1.000 (1.000, 1.001) | | <0.01 | 0.634 | 0.996 (0.993, 0.998) | <0.01 | <0.001 | |
|  |  |  |  |  |  |  |  | |  |  |  |  |  | |
| **Emergency department presentations in year prior (per 10 presentations)** | 1.45 (1.43, 1.47) | 0.01 | <0.001 | 1.64 (1.59, 1.70) | 0.03 | <0.001 | 1.38 (1.360, 1.40) | | 0.01 | <0.001 | 1.23 (1.22, 1.24) | 0.01 | <0.001 | |
|  |  |  |  |  |  |  |  | |  |  |  |  |  | |
| **History of depression** | 0.98 (0.96, 0.99) | 0.01 | 0.01 | 1.52 (1.46, 1.58) | 0.03 | <0.001 | 1.09 (1.06, 1.11) | | 0.01 | <0.001 | 1.00 (0.98, 1.02) | 0.01 | 0.949 | |
|  |  |  |  |  |  |  |  | |  |  |  |  |  | |
| **History of drug or alcohol abuse** | 1.02 (1.00, 1.05) | 0.01 | 0.027 | 0.95 (0.91, 1.00) | 0.02 | 0.059 | 0.84 (0.81, 0.87) | | 0.01 | <0.001 | 1.2 (1.17, 1.22) | 0.01 | <0.001 | |
|  |  |  |  |  |  |  |  | |  |  |  |  |  | |
| **History of psychotic disorder** | 0.66 (0.64, 0.67) | 0.01 | <0.001 | 1.36 (1.30, 1.42) | 0.03 | <0.001 | 1.27 (1.24, 1.30) | | 0.02 | <0.001 | 0.82 (0.80, 0.84) | 0.01 | <0.001 | |
|  |  |  |  |  |  |  |  | |  |  |  |  |  | |
| **History of anxiety disorder** | 1.00 (0.98, 1.02) | 0.01 | 0.689 | 2.30 (2.21, 2.39) | 0.05 | <0.001 | 1.30 (1.27, 1.33) | | 0.02 | <0.001 | 1.13 (1.11, 1.16) | 0.01 | <0.001 | |
|  |  |  |  |  |  |  |  | |  |  |  |  |  | |
| **History of delirium** | 1.33 (1.32, 1.35) | 0.01 | <0.001 | 2.15 (2.08, 2.22) | 0.04 | <0.001 | 1.01 (0.99, 1.03) | | 0.01 | 0.463 | 1.08 (1.07, 1.10) | 0.01 | <0.001 | |
|  |  |  |  |  |  |  |  | |  |  |  |  |  | |
| **History of behavioural problems** | 1.07 (1.06, 1.09) | 0.01 | <0.001 | 1.24 (1.19, 1.29) | 0.03 | <0.001 | 1.09 (1.07, 1.12) | | 0.01 | <0.001 | 0.99 (0.97, 1.01) | 0.01 | 0.211 | |
|  |  |  |  |  |  |  |  | |  |  |  |  |  | |
| **History of personality disorders** | 0.81 (0.77, 0.84) | 0.02 | <0.001 | 0.89 (0.83, 0.96) | 0.03 | 0.001 | 1 (0.96, 1.04) | | 0.02 | 0.985 | 1.01 (0.97, 1.05) | 0.02 | 0.641 | |
| * Wald test for variable inclusion | | | | | | | | | | | | | | |
| ^✝^ “Unknown” category excluded from output | | | | | | | |  | | | | | |  |

## Table S3: Adjusted incidence rate ratio's (aIRRs) for service use for the self-harm cohort, full regression results, reported separately after entry into the dementia and self-harm subgroup (without mild cognitive impairment as an indicator of dementia)

|  | **Hospital Admissions (Physical health)** | | | **Hospital Admissions (Mental health)** | | | **Mental Health Ambulatory** | | | | **Emergency Department Presentations** | | | | |
| --- | --- | --- | --- | --- | --- | --- | --- | --- | --- | --- | --- | --- | --- | --- | --- |
| **Variable** | **Incident Rate Ratio (95% CI)** | **Standard Error** | **p value** | **Incident Rate Ratio (95% CI)** | **Standard Error** | **p value** | **Incident Rate Ratio (95% CI)** | | **Standard Error** | **p value** | **Incident Rate Ratio (95% CI)** | **Standard Error** | **p value** | | |
| **Cohort membership** |  |  |  |  |  |  |  | |  |  |  |  |  | | |
| Self-harm only | 1.00 | … | … | 1.00 | … | … | 1.00 | | … | … | 1.00 | … | … | | |
| Dementia and self-harm | 1.24 (1.18, 1.30) | 0.03 | <0.001 | 0.90 (0.83, 0.98) | 0.04 | 0.016 | 0.98 (0.95, 1.01) | | 0.01 | 0.16 | 0.79 (0.75, 0.83) | 0.02 | <0.001 | | |
|  |  |  |  |  |  |  |  | |  |  |  |  |  | | |
| **Age** |  |  |  |  |  |  |  | |  |  |  |  |  | | |
| Linear | 0.98 (0.97, 0.98) | <0.01 | <0.001 | 0.80 (0.79, 0.81) | 0.01 | <0.001 | 0.87 (0.86, 0.87) | | <0.01 | <0.001 | 0.89 (0.88, 0.89) | <0.01 | <0.001 | | |
| Quadratic | 1.00 (1.00, 1.00) | <0.01 | <0.001 | 1.00 (1.00, 1.00) | <0.01 | <0.001 | 1.00 (1.00, 1.00) | | <0.01 | <0.001 | 1.00 (1.00, 1.00) | <0.01 | <0.001 | | |
|  |  |  |  |  |  |  |  | |  |  |  |  |  | | |
| **Sex (Female)**^✝^ | 0.96 (0.93, 0.99) | 0.01 | 0.005 | 0.99 (0.94, 1.05) | 0.03 | 0.757 | 0.95 (0.9, 1.00) | | 0.03 | 0.06 | 0.90 (0.87, 0.92) | 0.01 | <0.001 | | |
|  |  |  |  |  |  |  |  | |  |  |  |  |  | | |
| **Remoteness**^✝^ |  |  | <0.012* |  |  | <0.001* |  | |  | <0.618* |  |  | <0.001* | | |
| Major cities (Reference) | 1.00 | … | … | 1.00 | … | … | 1.00 | | … | … | 1.00 | … | … | | |
| Inner Regional | 1.05 (1.01, 1.09) | 0.02 | 0.006 | 0.97 (0.91, 1.04) | 0.03 | 0.342 | 1.03 (0.96, 1.10) | | 0.04 | 0.359 | 1.12 (1.08, 1.16) | 0.02 | <0.001 | | |
| Outer regional and beyond | 1.06 (1.00, 1.12) | 0.03 | 0.071 | 0.79 (0.71, 0.88) | 0.04 | <0.001 | 0.99 (0.89, 1.11) | | 0.05 | 0.914 | 1.02 (0.96, 1.08) | 0.03 | 0.517 | | |
|  |  |  |  |  |  |  |  | |  |  |  |  |  | | |
| **Index of relative socioeconomic disadvantage quintile**^✝^ |  |  | <0.001* |  |  | <0.001* |  | |  | <0.001* |  |  | <0.001* | | |
| 1-2 (Most disadvantaged) | 1 | … | … | 1 | … | … | 1 | | … | … | 1 | … | … | | |
| 3-4 | 0.97 (0.93, 1.01) | 0.02 | 0.146 | 1.20 (1.11, 1.29) | 0.04 | <0.001 | 1.16 (1.08, 1.24) | | 0.04 | <0.001 | 1.04 (1.00, 1.09) | 0.02 | 0.028 | | |
| 5-6 | 0.88 (0.84, 0.92) | 0.02 | <0.001 | 1.37 (1.26, 1.48) | 0.06 | <0.001 | 0.99 (0.91, 1.08) | | 0.04 | 0.867 | 0.96 (0.92, 1.01) | 0.02 | 0.096 | | |
| 7-8 | 0.97 (0.92, 1.02) | 0.03 | 0.268 | 1.79 (1.62, 1.97) | 0.09 | <0.001 | 1.16 (1.05, 1.28) | | 0.06 | 0.003 | 0.91 (0.86, 0.95) | 0.02 | <0.001 | | |
| 9-10 (Least disadvantaged) | 1.00 (0.95, 1.05) | 0.03 | 0.892 | 2.35 (2.14, 2.58) | 0.11 | <0.001 | 1.14 (1.04, 1.26) | | 0.06 | 0.006 | 0.83 (0.78, 0.87) | 0.02 | <0.001 | | |
|  |  |  |  |  |  |  |  | |  |  |  |  |  | | |
| **Num. Elixhauser comorbidities in year prior** | 1.11 (1.10, 1.11) | <0.01 | <0.001 | 1.01 (1.00, 1.02) | 0.01 | 0.021 | 1.01 (1.01, 1.02) | | <0.01 | <0.001 | 1.06 (1.05, 1.07) | <0.01 | <0.001 | | |
|  |  |  |  |  |  |  |  | |  |  |  |  |  | | |
| **MH Ambulatory use in year prior  (per 10 days)** | 1.00 (1.00, 1.01) | <0.01 | 0.004 | 0.99 (0.99, 1) | <0.01 | 0.003 | 1.04 (1.04, 1.04) | | <0.01 | <0.001 | 1.01 (1.01, 1.01) | <0.01 | <0.001 | | |
|  |  |  |  |  |  |  |  | |  |  |  |  |  | | |
| **Involuntary mental health admissions in year prior (per 10 admissions)** | 1.00 (0.99, 1.00) | <0.01 | 0.232 | 0.99 (0.99, 1.00) | <0.01 | 0.006 | 1.01 (1.01, 1.01) | | <0.01 | <0.001 | 0.99 (0.99, 1.00) | <0.01 | 0.001 | | |
|  |  |  |  |  |  |  |  | |  |  |  |  |  | | |
| **Emergency department presentations in year prior (per 10 presentations)** | 1.13 (1.12, 1.15) | 0.01 | <0.001 | 1.18 (1.16, 1.20) | 0.01 | <0.001 | 1.08 (1.07, 1.09) | | <0.01 | <0.001 | 1.14 (1.14, 1.15) | <0.01 | <0.001 | | |
|  |  |  |  |  |  |  |  | |  |  |  |  |  | | |
| **History of depression** | 0.94 (0.91, 0.96) | 0.01 | <0.001 | 2.7 (2.58, 2.83) | 0.06 | <0.001 | 0.99 (0.97, 1.00) | | 0.01 | 0.106 | 0.86 (0.84, 0.88) | 0.01 | <0.001 | | |
|  |  |  |  |  |  |  |  | |  |  |  |  |  | | |
| **History of drug or alcohol abuse** | 0.90 (0.88, 0.93) | 0.01 | <0.001 | 1.64 (1.57, 1.71) | 0.04 | <0.001 | 1.40 (1.38, 1.43) | | 0.01 | <0.001 | 1.10 (1.07, 1.13) | 0.02 | <0.001 | | |
|  |  |  |  |  |  |  |  | |  |  |  |  |  | | |
| **History of psychotic disorder** | 0.94 (0.91, 0.96) | 0.01 | <0.001 | 2.70 (2.58, 2.83) | 0.06 | <0.001 | 0.99 (0.97, 1.00) | | 0.01 | 0.106 | 0.86 (0.84, 0.88) | 0.01 | <0.001 | | |
|  |  |  |  |  |  |  |  | |  |  |  |  |  | | |
| **History of anxiety disorder** | 1.09 (1.06, 1.12) | 0.01 | <0.001 | 1.27 (1.21, 1.32) | 0.03 | <0.001 | 0.90 (0.89, 0.92) | | 0.01 | <0.001 | 1.28 (1.25, 1.31) | 0.02 | <0.001 | | |
|  |  |  |  |  |  |  |  | |  |  |  |  |  | | |
| **History of delirium** | 0.84 (0.82, 0.86) | 0.01 | <0.001 | 1.37 (1.32, 1.43) | 0.03 | <0.001 | 1.12 (1.10, 1.14) | | 0.01 | <0.001 | 0.89 (0.87, 0.91) | 0.01 | <0.001 | | |
|  |  |  |  |  |  |  |  | |  |  |  |  |  | | |
| **History of behavioural problems** | 1.24 (1.19, 1.29) | 0.02 | <0.001 | 1.11 (1.05, 1.18) | 0.03 | 0.001 | 1.04 (1.02, 1.06) | | 0.01 | <0.001 | 1.02 (0.98, 1.05) | 0.02 | 0.35 | | |
|  |  |  |  |  |  |  |  | |  |  |  |  |  | | |
| **History of personality disorders** | 1.02 (0.99, 1.05) | 0.01 | 0.12 | 1.13 (1.09, 1.17) | 0.02 | <0.001 | 1.30 (1.28, 1.32) | | 0.01 | <0.001 | 1.08 (1.06, 1.11) | 0.01 | <0.001 | | |
| * Wald test for variable inclusion | | | | | | | | | | | | | | |  |
| ^✝^ “Unknown” category excluded from output | | | | | | | |  | | | | | |  |  |

## 2.2 Dementia and self-harm subgroup

## Table S4: Rates of service use per 1,000 person years for the dementia and self-harm subgroup only (without mild cognitive impairment as an indicator of dementia). Number of events not included to prevent risk of re-identification.

|  | **One year prior to subgroup entry** | | **One year after subgroup entry** | | **More than one year after subgroup entry** | |
| --- | --- | --- | --- | --- | --- | --- |
| **Service-use type** | **Dementia cohort*** | **Self-harm cohort** | **Dementia cohort*** | **Self-harm cohort** | **Dementia cohort*** | **Self-harm cohort** |
| **Hospital admissions  (physical health)** |  |  |  |  |  |  |
| Rate (per 1000 person years) | 2764.0 | 2934.7 | 3279.4 | 3895.8 | 1356.2 | 3026.7 |
| **Hospital admissions  (mental health)** |  |  |  |  |  |  |
| Rate (per 1000 person years) | 1233.9 | 930.5 | 1396.7 | 1259.229 | 458.2 | 434.9 |
| **Mental health ambulatory days** |  |  |  |  |  |  |
| Rate (per 1000 person years) | 6052.6 | 7019.8 | 8818.5 | 8971.045 | 5621.3 | 5222.9 |
| **Emergency department presentations** |  |  |  |  |  |  |
| Rate (per 1000 person years) | 3756.9 | 4900.7 | 3756.3 | 3311.4 | 1885.8 | 2274.8 |
| *Excludes people who had their first self-harm diagnosis on the same day as their entry to the dementia cohort, *n* = 206 | | | | | | |

## Table S5: Predictors of service use for the dementia and self-harm cohort (without mild cognitive impairment as an indicator of dementia)

|  | **Hospital Admissions (Physical health)** | | | **Hospital Admissions (Mental health)** | | | **Mental Health Ambulatory** | | | | **Emergency Department Presentations** | | | |
| --- | --- | --- | --- | --- | --- | --- | --- | --- | --- | --- | --- | --- | --- | --- |
| **Variable** | **Incident Rate Ratio (95% CI)** | **Standard Error** | **p value** | **Incident Rate Ratio (95% CI)** | **Standard Error** | **p value** | **Incident Rate Ratio (95% CI)** | | **Standard Error** | **p value** | **Incident Rate Ratio (95% CI)** | **Standard Error** | **p value** | |
| **Age** |  |  |  |  |  |  |  | |  |  |  |  |  | |
| Linear | 1.01 (0.97, 1.05) | 0.02 | 0.629 | 0.91 (0.85, 0.97) | 0.03 | 0.005 | 1.45 (1.39, 1.50) | | 0.03 | <0.001 | 0.91 (0.88, 0.94) | 0.02 | <0.001 | |
| Quadratic | 1.000 (1.000, 1.000) | 0.021 | 0.317 | 1.000 (1.000, 1.001) | 0.03 | 0.122 | 0.996 (0.996, 0.997) | | 0.028 | <0.001 | 1.001 (1, 1.001) | 0.02 | <0.001 | |
|  |  |  |  |  |  |  |  | |  |  |  |  |  | |
| **Sex (Female)**^✝^ | 0.78 (0.68, 0.89) | 0.05 | <0.001 | 0.90 (0.74, 1.10) | 0.09 | 0.314 | 0.89 (0.71, 1.12) | | 0.10 | 0.332 | 0.75 (0.66, 0.86) | 0.05 | <0.001 | |
|  |  |  |  |  |  |  |  | |  |  |  |  |  | |
| **Remoteness**^✝^ |  |  | 0.162* |  |  | <0.001* |  | |  | 0.033* |  |  | 0.111* | |
| Major cities (Reference) | 1 | … | … | 1 | … | … | 1 | | … | … | 1 | … | … | |
| Inner Regional | 0.99 (0.83, 1.18) | 0.09 | 0.943 | 0.68 (0.53, 0.89) | 0.09 | 0.004 | 0.74 (0.55, 0.99) | | 0.11 | 0.043 | 0.91 (0.76, 1.09) | 0.08 | 0.297 | |
| Outer regional and beyond | 0.75 (0.56, 1.01) | 0.11 | 0.057 | 1.59 (1.04, 2.41) | 0.34 | 0.031 | 1.43 (0.87, 2.33) | | 0.36 | 0.157 | 0.74 (0.55, 1.00) | 0.11 | 0.053 | |
|  |  |  |  |  |  |  |  | |  |  |  |  |  | |
| **Index of relative socioeconomic disadvantage quintile**^✝^ |  |  | <0.001* |  |  | 0.248* |  | |  | 0.780* |  |  | 0.015* | |
| 1-2 (Most disadvantaged) | 1 | … | … | 1 | … | … | 1 | | … | … | 1 | … | … | |
| 3-4 | 0.76 (0.63, 0.92) | 0.07 | 0.004 | 0.97 (0.74, 1.27) | 0.13 | 0.807 | 1.07 (0.78, 1.46) | | 0.17 | 0.685 | 1.27 (1.05, 1.52) | 0.12 | 0.012 | |
| 5-6 | 0.67 (0.55, 0.83) | 0.07 | <0.001 | 1.07 (0.79, 1.45) | 0.17 | 0.671 | 0.90 (0.64, 1.28) | | 0.16 | 0.566 | 1.14 (0.93, 1.40) | 0.12 | 0.200 | |
| 7-8 | 0.96 (0.77, 1.20) | 0.11 | 0.721 | 1.16 (0.83, 1.62) | 0.20 | 0.386 | 0.84 (0.58, 1.24) | | 0.16 | 0.384 | 0.88 (0.70, 1.11) | 0.10 | 0.277 | |
| 9-10 (Least disadvantaged) | 0.69 (0.56, 0.85) | 0.07 | 0.001 | 1.34 (0.99, 1.81) | 0.21 | 0.058 | 0.99 (0.69, 1.40) | | 0.18 | 0.945 | 1.15 (0.93, 1.42) | 0.12 | 0.189 | |
|  |  |  |  |  |  |  |  | |  |  |  |  |  | |
| **Num. Elixhauser comorbidities in year prior** | 1.04 (1.03, 1.05) | 0.01 | <0.001 | 1.08 (1.04, 1.11) | 0.02 | <0.001 | 1.02 (1.00, 1.03) | | 0.01 | 0.015 | 1.07 (1.06, 1.09) | 0.01 | <0.001 | |
|  |  |  |  |  |  |  |  | |  |  |  |  |  | |
| **MH Ambulatory use in year prior  (per 10 days)** | 0.97 (0.96, 0.99) | 0.01 | 0.002 | 0.95 (0.93, 0.97) | 0.01 | <0.001 | 1.01 (1.00, 1.01) | | <0.01 | <0.001 | 1.00 (0.98, 1.01) | 0.01 | 0.546 | |
|  |  |  |  |  |  |  |  | |  |  |  |  |  | |
| **Involuntary mental health admissions in year prior (per 10 admissions)** | 1.00 (0.99, 1.01) | <0.01 | 0.618 | 1.02 (1.01, 1.03) | 0.01 | <0.001 | 1.00 (1.00, 1.00) | | <0.01 | 0.127 | 1.00 (1.00, 1.01) | <0.01 | 0.292 | |
|  |  |  |  |  |  |  |  | |  |  |  |  |  | |
| **Emergency department presentations in year prior (per 10 presentations)** | 1.06 (1.02, 1.10) | 0.02 | 0.001 | 1.08 (1.03, 1.14) | 0.03 | 0.003 | 1.02 (0.99, 1.05) | | 0.02 | 0.165 | 1.12 (1.09, 1.14) | 0.01 | <0.001 | |
|  |  |  |  |  |  |  |  | |  |  |  |  |  | |
| **History of depression** | 0.92 (0.82, 1.04) | 0.06 | 0.184 | 1.58 (1.31, 1.91) | 0.15 | <0.001 | 0.97 (0.87, 1.07) | | 0.05 | 0.508 | 0.95 (0.86, 1.07) | 0.05 | 0.411 | |
|  |  |  |  |  |  |  |  | |  |  |  |  |  | |
| **History of drug or alcohol abuse** | 0.82 (0.71, 0.94) | 0.06 | 0.003 | 0.94 (0.75, 1.17) | 0.11 | 0.591 | 0.53 (0.47, 0.61) | | 0.04 | <0.001 | 1.26 (1.10, 1.44) | 0.09 | 0.001 | |
|  |  |  |  |  |  |  |  | |  |  |  |  |  | |
| **History of psychotic disorder** | 0.76 (0.68, 0.85) | 0.04 | <0.001 | 1.05 (0.88, 1.26) | 0.09 | 0.573 | 0.78 (0.7, 0.87) | | 0.04 | <0.001 | 0.63 (0.57, 0.7) | 0.03 | <0.001 | |
|  |  |  |  |  |  |  |  | |  |  |  |  |  | |
| **History of anxiety disorder** | 1.05 (0.96, 1.15) | 0.05 | 0.284 | 1.21 (1.01, 1.44) | 0.11 | 0.036 | 0.93 (0.84, 1.03) | | 0.05 | 0.159 | 1.40 (1.26, 1.54) | 0.07 | <0.001 | |
|  |  |  |  |  |  |  |  | |  |  |  |  |  | |
| **History of delirium** | 1.02 (0.91, 1.14) | 0.06 | 0.72 | 1.26 (1.06, 1.50) | 0.11 | 0.008 | 1.04 (0.95, 1.15) | | 0.05 | 0.405 | 0.79 (0.71, 0.87) | 0.04 | <0.001 | |
|  |  |  |  |  |  |  |  | |  |  |  |  |  | |
| **History of behavioural problems** | 0.94 (0.87, 1.01) | 0.04 | 0.094 | 1.25 (1.07, 1.47) | 0.1 | 0.005 | 1.03 (0.95, 1.12) | | 0.04 | 0.437 | 0.89 (0.82, 0.97) | 0.04 | 0.01 | |
|  |  |  |  |  |  |  |  | |  |  |  |  |  | |
| **History of personality disorders** | 0.88 (0.77, 1.01) | 0.06 | 0.065 | 0.86 (0.71, 1.04) | 0.08 | 0.121 | 0.99 (0.88, 1.12) | | 0.06 | 0.924 | 1.14 (1.03, 1.27) | 0.06 | 0.014 | |
| * Wald test for variable inclusion | | | | | | | | | | | | | | |
| ^✝^ “Unknown” category excluded from output | | | | | | | |  | | | | | |  |

## Table S6: Predictors of time to re-presentation to a hospital episode or emergency department for the dementia and self-harm subgroup after first date of entry to the subgroup (without mild cognitive impairment as an indicator of dementia)

|  | **Emergency department** | | | **Hospital episodes** | | | | |
| --- | --- | --- | --- | --- | --- | --- | --- | --- |
| **Variable** | **Hazard Ratio (95% CI)** | **Standard Error** | **p value** | **Hazard Ratio (95% CI)** | | **Standard Error** | **p value** | |
| **Age** |  |  |  |  | |  |  | |
| Linear | 0.86 (0.77, 0.95) | 0.05 | 0.004 | 0.86 (0.77, 0.97) | | 0.05 | 0.011 | |
| Quadratic | 1.001 (1.000, 1.002) | <0.01 | 0.01 | 1.001 (1.000, 1.002) | | <0.01 | 0.026 | |
|  |  |  |  |  | |  |  | |
| **Sex (Female)** | 0.82 (0.55, 1.22) | 0.17 | 0.333 | 1.52 (0.47, 4.94) | | 0.91 | 0.488 | |
|  |  |  |  |  | |  |  | |
| **Remoteness**^✝^ |  |  | 0.985* |  | |  | 0.124* | |
| Major cities (Reference) | 1.00 | … | … | 1.00 | | … | … | |
| Inner Regional | 1.02 (0.68, 1.52) | 0.21 | 0.93 | 1.39 (0.88, 2.18) | | 0.32 | 0.16 | |
| Outer regional and beyond | 1.05 (0.58, 1.91) | 0.32 | 0.863 | 1.82 (0.96, 3.42) | | 0.59 | 0.065 | |
|  |  |  |  |  | |  |  | |
| **Index of relative socioeconomic disadvantage quintile**^✝^ |  |  | 0.170* |  | |  | 0.526* | |
| 1-2 (Most disadvantaged) | 1.00 | … | … | 1.00 | | … | … | |
| 3-4 | 0.76 (0.51, 1.13) | 0.15 | 0.173 | 1.13 (0.74, 1.73) | | 0.25 | 0.574 | |
| 5-6 | 0.99 (0.35, 2.77) | 0.52 | 0.986 | 0.11 (0.02, 0.69) | | 0.1 | 0.019 | |
| 7-8 | 0.40 (0.17, 0.95) | 0.18 | 0.037 | 0.55 (0.20, 1.53) | | 0.29 | 0.253 | |
| 9-10 (Least disadvantaged) | 0.75 (0.49, 1.15) | 0.16 | 0.188 | 0.94 (0.56, 1.59) | | 0.25 | 0.813 | |
|  |  |  |  |  | |  |  | |
| **EH Comorbidities in year prior** | 1.30 (1.21, 1.39) | 0.05 | <0.001 | 1.33 (1.24, 1.43) | | 0.05 | <0.001 | |
|  |  |  |  |  | |  |  | |
| **MH Ambulatory use in year prior (per 10 days)** | 0.89 (0.76, 1.04) | 0.07 | 0.134 | 0.85 (0.73, 0.99) | | 0.06 | 0.036 | |
|  |  |  |  |  | |  |  | |
| **Involuntary MH admissions in year prior (per 10 admissions)** | 0.96 (0.88, 1.04) | 0.04 | 0.315 | 1.01 (0.98, 1.04) | | 0.01 | 0.439 | |
|  |  |  |  |  | |  |  | |
| **ED presentations in year prior (per 10 presentations)** | 1.06 (1.04, 1.08) | 0.01 | <0.001 | 1.05 (1.02, 1.07) | | 0.01 | <0.001 | |
|  |  |  |  |  | |  |  | |
| **History of depression** | 0.93 (0.67, 1.30) | 0.16 | 0.685 | 1.24 (0.88, 1.75) | | 0.22 | 0.219 | |
|  |  |  |  |  | |  |  | |
| **History of drug or alcohol abuse** | 1.32 (0.93, 1.85) | 0.23 | 0.116 | 1.42 (1.01, 2.01) | | 0.25 | 0.044 | |
|  |  |  |  |  | |  |  | |
| **History of psychotic disorder** | 1.03 (0.76, 1.39) | 0.16 | 0.854 | 1.11 (0.8, 1.55) | | 0.19 | 0.528 | |
|  |  |  |  |  | |  |  | |
| **History of anxiety disorder** | 0.91 (0.67, 1.23) | 0.14 | 0.539 | 0.66 (0.48, 0.91) | | 0.11 | 0.011 | |
|  |  |  |  |  | |  |  | |
| **History of delirium** | 1.53 (1.07, 2.19) | 0.28 | 0.021 | 1.16 (0.78, 1.72) | | 0.23 | 0.465 | |
|  |  |  |  |  | |  |  | |
| **History of behavioural problems** | 1.21 (0.89, 1.63) | 0.18 | 0.223 | 1.22 (0.89, 1.67) | | 0.2 | 0.225 | |
|  |  |  |  |  | |  |  | |
| **History of personality disorders** | 0.74 (0.49, 1.13) | 0.16 | 0.163 | 0.75 (0.47, 1.18) | | 0.17 | 0.208 | |
| * Wald test for variable inclusion | | | | | | | | |
| ^✝^ “Unknown” category excluded from output | | | | |  | | |  |

# 3 Full regression results for service use

## Table S7: Adjusted incidence rate ratio's (aIRRs) for service use for the dementia cohort, full regression results, reported separately after entry into the dementia and self-harm subgroup

|  | **Hospital Admissions (Physical health)** | | | **Hospital Admissions (Mental health)** | | | **Mental Health Ambulatory** | | | | **Emergency Department Presentations** | | | |
| --- | --- | --- | --- | --- | --- | --- | --- | --- | --- | --- | --- | --- | --- | --- |
| **Variable** | **Incident Rate Ratio (95% CI)** | **Standard Error** | **p value** | **Incident Rate Ratio (95% CI)** | **Standard Error** | **p value** | **Incident Rate Ratio (95% CI)** | | **Standard Error** | **p value** | **Incident Rate Ratio (95% CI)** | **Standard Error** | **p value** | |
| **Cohort membership** |  |  |  |  |  |  |  | |  |  |  |  |  | |
| Dementia only | 1.00 | … | … | 1.00 | … | … | 1.00 | | … | … | 1.00 | … | … | |
| Dementia and self-harm | 0.96 (0.9, 1.01) | 0.03 | 0.125 | 0.57 (0.51, 0.63) | 0.03 | <0.001 | 1.33 (1.17, 1.51) | | 0.09 | <0.001 | 0.86 (0.81, 0.91) | 0.02 | <0.001 | |
|  |  |  |  |  |  |  |  | |  |  |  |  |  | |
| **Age** |  |  |  |  |  |  |  | |  |  |  |  |  | |
| Linear | 1.17 (1.17, 1.18) | <0.01 | <0.001 | 1.02 (1, 1.03) | 0.01 | 0.029 | 1.15 (1.14, 1.17) | | 0.01 | <0.001 | 1.01 (1, 1.01) | <0.01 | 0.11 | |
| Quadratic | 0.999 (0.999, 0.999) | <0.01 | <0.001 | 1.000 (1.000, 1.000) | <0.01 | <0.001 | 0.999 (0.999, 0.999) | | <0.01 | <0.001 | 1.000 (1.000, 1.000) | <0.01 | 0.027 | |
|  |  |  |  |  |  |  |  | |  |  |  |  |  | |
| **Sex (Female)**^✝^ | 0.72 (0.71, 0.73) | 0.01 | <0.001 | 0.66 (0.64, 0.68) | 0.01 | <0.001 | 0.90 (0.86, 0.94) | | 0.02 | <0.001 | 0.71 (0.70, 0.72) | 0.01 | <0.001 | |
|  |  |  |  |  |  |  |  | |  |  |  |  |  | |
| **Remoteness**^✝^ |  |  | <0.001* |  |  | <0.303* |  | |  | <0.001* |  |  | <0.001* | |
| Major cities (Reference) | 1.00 | … | … | 1.00 | … | … | 1.00 | | … | … | 1.00 | … | … | |
| Inner Regional | 0.89 (0.88, 0.91) | 0.01 | <0.001 | 1 (0.96, 1.04) | 0.02 | 0.936 | 1.6 (1.52, 1.69) | | 0.04 | <0.001 | 0.79 (0.78, 0.81) | 0.01 | <0.001 | |
| Outer regional and beyond | 0.93 (0.90, 0.96) | 0.01 | <0.001 | 1.05 (0.98, 1.13) | 0.04 | 0.135 | 1.62 (1.5, 1.76) | | 0.07 | <0.001 | 0.68 (0.66, 0.70) | 0.01 | <0.001 | |
|  |  |  |  |  |  |  |  | |  |  |  |  |  | |
| **Index of relative socioeconomic disadvantage quintile**^✝^ |  |  | <0.001* |  |  | <0.001* |  | |  | <0.001* |  |  | <0.001* | |
| 1-2 (Most disadvantaged) | 1.00 | … | … | 1.00 | … | … | 1.00 | | … | … | 1.00 | … | … | |
| 3-4 | 0.91 (0.89, 0.93) | 0.01 | <0.001 | 0.89 (0.85, 0.93) | 0.02 | <0.001 | 1.24 (1.17, 1.31) | | 0.04 | <0.001 | 0.89 (0.88, 0.91) | 0.01 | <0.001 | |
| 5-6 | 0.92 (0.90, 0.94) | 0.01 | <0.001 | 0.92 (0.87, 0.97) | 0.02 | 0.001 | 1.21 (1.13, 1.29) | | 0.04 | <0.001 | 0.87 (0.86, 0.89) | 0.01 | <0.001 | |
| 7-8 | 0.93 (0.90, 0.95) | 0.01 | <0.001 | 1.07 (1.01, 1.13) | 0.03 | 0.022 | 1.42 (1.32, 1.52) | | 0.05 | <0.001 | 0.85 (0.83, 0.87) | 0.01 | <0.001 | |
| 9-10 (Least disadvantaged) | 0.88 (0.86, 0.9) | 0.01 | <0.001 | 0.88 (0.83, 0.92) | 0.02 | <0.001 | 1.25 (1.16, 1.34) | | 0.04 | <0.001 | 0.70 (0.68, 0.72) | 0.01 | <0.001 | |
|  |  |  |  |  |  |  |  | |  |  |  |  |  | |
| **Num. Elixhauser comorbidities in year prior** | 1.00 (1.00, 1.00) | <0.01 | 0.414 | 0.95 (0.94, 0.96) | <0.01 | <0.001 | 0.97 (0.96, 0.99) | | 0.01 | <0.001 | 1.04 (1.03, 1.04) | <0.01 | <0.001 | |
|  |  |  |  |  |  |  |  | |  |  |  |  |  | |
| **MH Ambulatory use in year prior  (per 10 days)** | 1.02 (1.013, 1.027) | <0.01 | <0.001 | 0.991 (0.981, 1.001) | 0.01 | 0.085 | 1.005 (1.003, 1.007) | | <0.01 | <0.001 | 0.999 (0.992, 1.006) | <0.01 | 0.739 | |
|  |  |  |  |  |  |  |  | |  |  |  |  |  | |
| **Involuntary mental health admissions in year prior (per 10 admissions)** | 0.992 (0.989, 0.995) | <0.01 | <0.001 | 0.993 (0.990, 0.995) | <0.01 | <0.001 | 1.088 (1.084, 1.093) | | <0.01 | <0.001 | 0.996 (0.993, 0.998) | <0.01 | <0.001 | |
|  |  |  |  |  |  |  |  | |  |  |  |  |  | |
| **Emergency department presentations in year prior (per 10 presentations)** | 1.45 (1.43, 1.47) | 0.01 | <0.001 | 1.64 (1.59, 1.7) | 0.03 | <0.001 | 1.57 (1.49, 1.65) | | 0.04 | <0.001 | 1.23 (1.22, 1.24) | 0.01 | <0.001 | |
|  |  |  |  |  |  |  |  | |  |  |  |  |  | |
| **History of depression** | 0.97 (0.96, 0.99) | 0.01 | 0.006 | 1.52 (1.46, 1.58) | 0.03 | <0.001 | 1.63 (1.55, 1.71) | | 0.04 | <0.001 | 1.00 (0.98, 1.02) | 0.01 | 0.952 | |
|  |  |  |  |  |  |  |  | |  |  |  |  |  | |
| **History of drug or alcohol abuse** | 1.03 (1, 1.05) | 0.01 | 0.019 | 0.95 (0.91, 1.00) | 0.02 | 0.054 | 0.66 (0.62, 0.70) | | 0.02 | <0.001 | 1.20 (1.17, 1.22) | 0.01 | <0.001 | |
|  |  |  |  |  |  |  |  | |  |  |  |  |  | |
| **History of psychotic disorder** | 0.65 (0.64, 0.67) | 0.01 | <0.001 | 1.37 (1.31, 1.43) | 0.03 | <0.001 | 4.73 (4.51, 4.96) | | 0.11 | <0.001 | 0.82 (0.80, 0.84) | 0.01 | <0.001 | |
|  |  |  |  |  |  |  |  | |  |  |  |  |  | |
| **History of anxiety disorder** | 1.00 (0.98, 1.02) | 0.01 | 0.872 | 2.30 (2.21, 2.4) | 0.05 | <0.001 | 2.06 (1.96, 2.16) | | 0.05 | <0.001 | 1.13 (1.11, 1.16) | 0.01 | <0.001 | |
|  |  |  |  |  |  |  |  | |  |  |  |  |  | |
| **History of delirium** | 1.34 (1.32, 1.35) | 0.01 | <0.001 | 2.15 (2.08, 2.22) | 0.04 | <0.001 | 1.06 (1.01, 1.11) | | 0.03 | 0.028 | 1.08 (1.07, 1.10) | 0.01 | <0.001 | |
|  |  |  |  |  |  |  |  | |  |  |  |  |  | |
| **History of behavioural problems** | 1.07 (1.05, 1.09) | 0.01 | <0.001 | 1.24 (1.19, 1.29) | 0.03 | <0.001 | 1.32 (1.26, 1.40) | | 0.04 | <0.001 | 0.99 (0.97, 1.01) | 0.01 | 0.21 | |
|  |  |  |  |  |  |  |  | |  |  |  |  |  | |
| **History of personality disorders** | 0.8 (0.77, 0.84) | 0.02 | <0.001 | 0.89 (0.83, 0.96) | 0.03 | 0.001 | 1.32 (1.21, 1.44) | | 0.06 | <0.001 | 1.01 (0.97, 1.05) | 0.02 | 0.641 | |
| * Wald test for variable inclusion | | | | | | | | | | | | | | |
| ^✝^ “Unknown” category excluded from output | | | | | | | |  | | | | | |  |

## Table S8: Adjusted incidence rate ratio's (aIRRs) for service use for the self-harm cohort, full regression results, reported separately after entry into the dementia and self-harm subgroup

|  | **Hospital Admissions (Physical health)** | | | **Hospital Admissions (Mental health)** | | | **Mental Health Ambulatory** | | | | **Emergency Department Presentations** | | | |
| --- | --- | --- | --- | --- | --- | --- | --- | --- | --- | --- | --- | --- | --- | --- |
| **Variable** | **Incident Rate Ratio (95% CI)** | **Standard Error** | **p value** | **Incident Rate Ratio (95% CI)** | **Standard Error** | **p value** | **Incident Rate Ratio (95% CI)** | | **Standard Error** | **p value** | **Incident Rate Ratio (95% CI)** | **Standard Error** | **p value** | |
| **Cohort membership** |  |  |  |  |  |  |  | |  |  |  |  |  | |
| Self-harm only | 1.00 | … | … | 1.00 | … | … | 1.00 | | … | … | 1.00 | … | … | |
| Dementia and self-harm | 1.24 (1.18, 1.30) | 0.03 | <0.001 | 0.90 (0.83, 0.98) | 0.04 | 0.015 | 0.96 (0.92, 0.99) | | 0.02 | 0.009 | 0.79 (0.75, 0.82) | 0.02 | <0.001 | |
|  |  |  |  |  |  |  |  | |  |  |  |  |  | |
| **Age** |  |  |  |  |  |  |  | |  |  |  |  |  | |
| Linear | 0.98 (0.97, 0.98) | <0.01 | <0.001 | 0.80 (0.79, 0.81) | 0.01 | <0.001 | 0.91 (0.90, 0.91) | | <0.01 | <0.001 | 0.89 (0.88, 0.89) | <0.01 | <0.001 | |
| Quadratic | 1.000 (1.000, 1.000) | <0.01 | <0.001 | 1.000 (1.000, 1.000) | <0.01 | <0.001 | 1.000 (1.000, 1.000) | | <0.01 | <0.001 | 1.000 (1.000, 1.000) | <0.01 | <0.001 | |
|  |  |  |  |  |  |  |  | |  |  |  |  |  | |
| **Sex (Female)**^✝^ | 0.96 (0.93, 0.99) | 0.01 | 0.006 | 0.99 (0.94, 1.05) | 0.03 | 0.75 | 0.89 (0.84, 0.94) | | 0.02 | <0.001 | 0.90 (0.87, 0.93) | 0.01 | <0.001 | |
|  |  |  |  |  |  |  |  | |  |  |  |  |  | |
| **Remoteness**^✝^ |  |  | <0.012* |  |  | <0.001* |  | |  | <0.618* |  |  | <0.001* | |
| Major cities (Reference) | 1.00 | … | … | 1.00 | … | … | 1.00 | | … | … | 1.00 | … | … | |
| Inner Regional | 1.05 (1.01, 1.09) | 0.02 | 0.006 | 0.97 (0.90, 1.03) | 0.03 | 0.329 | 1.01 (0.94, 1.08) | | 0.04 | 0.812 | 1.12 (1.08, 1.16) | 0.02 | <0.001 | |
| Outer regional and beyond | 1.06 (1.00, 1.12) | 0.03 | 0.07 | 0.79 (0.71, 0.88) | 0.04 | <0.001 | 0.90 (0.81, 1.01) | | 0.05 | 0.069 | 1.02 (0.96, 1.08) | 0.03 | 0.517 | |
|  |  |  |  |  |  |  |  | |  |  |  |  |  | |
| **Index of relative socioeconomic disadvantage quintile**^✝^ |  |  | <0.001* |  |  | <0.001* |  | |  | <0.001* |  |  | <0.001* | |
| 1-2 (Most disadvantaged) | 1.00 | … | … | 1.00 | … | … | 1.00 | | … | … | 1.00 | … | … | |
| 3-4 | 0.97 (0.93, 1.01) | 0.02 | 0.15 | 1.20 (1.11, 1.29) | 0.04 | <0.001 | 1.09 (1.02, 1.17) | | 0.04 | 0.018 | 1.04 (1.01, 1.09) | 0.02 | 0.027 | |
| 5-6 | 0.88 (0.84, 0.92) | 0.02 | <0.001 | 1.36 (1.26, 1.48) | 0.06 | <0.001 | 0.94 (0.87, 1.03) | | 0.04 | 0.178 | 0.96 (0.92, 1.01) | 0.02 | 0.102 | |
| 7-8 | 0.97 (0.92, 1.02) | 0.03 | 0.278 | 1.79 (1.62, 1.97) | 0.09 | <0.001 | 0.99 (0.89, 1.09) | | 0.05 | 0.803 | 0.91 (0.86, 0.95) | 0.02 | <0.001 | |
| 9-10 (Least disadvantaged) | 1.00 (0.95, 1.05) | 0.03 | 0.895 | 2.35 (2.14, 2.58) | 0.11 | <0.001 | 0.94 (0.85, 1.03) | | 0.05 | 0.18 | 0.83 (0.78, 0.87) | 0.02 | <0.001 | |
|  |  |  |  |  |  |  |  | |  |  |  |  |  | |
| **Num. Elixhauser comorbidities in year prior** | 1.11 (1.1, 1.11) | <0.01 | <0.001 | 1.01 (1, 1.02) | 0.01 | 0.022 | 1.01 (1, 1.01) | | <0.01 | 0.01 | 1.06 (1.06, 1.07) | <0.01 | <0.001 | |
|  |  |  |  |  |  |  |  | |  |  |  |  |  | |
| **MH Ambulatory use in year prior  (per 10 days)** | 1.008 (1.004, 1.011) | <0.01 | <0.001 | 0.992 (0.987, 0.997) | <0.01 | 0.001 | 1.037 (1.036, 1.037) | | <0.01 | <0.001 | 1.007 (1.004, 1.009) | <0.01 | <0.001 | |
|  |  |  |  |  |  |  |  | |  |  |  |  |  | |
| **Involuntary mental health admissions in year prior (per 10 admissions)** | 0.998 (0.995, 1.001) | <0.01 | 0.245 | 0.994 (0.989, 0.998) | <0.01 | 0.005 | 1.008 (1.007, 1.009) | | <0.01 | <0.001 | 0.995 (0.992, 0.998) | <0.01 | 0.003 | |
|  |  |  |  |  |  |  |  | |  |  |  |  |  | |
| **Emergency department presentations in year prior (per 10 presentations)** | 1.13 (1.12, 1.15) | 0.01 | <0.001 | 1.18 (1.16, 1.2) | 0.01 | <0.001 | 1.08 (1.08, 1.09) | | <0.01 | <0.001 | 1.14 (1.14, 1.15) | <0.01 | <0.001 | |
|  |  |  |  |  |  |  |  | |  |  |  |  |  | |
| **History of depression** | 0.94 (0.91, 0.96) | 0.01 | <0.001 | 2.70 (2.58, 2.83) | 0.06 | <0.001 | 0.98 (0.96, 1.00) | | 0.01 | 0.039 | 0.86 (0.84, 0.88) | 0.01 | <0.001 | |
|  |  |  |  |  |  |  |  | |  |  |  |  |  | |
| **History of drug or alcohol abuse** | 0.90 (0.88, 0.93) | 0.01 | <0.001 | 1.64 (1.57, 1.71) | 0.04 | <0.001 | 1.50 (1.47, 1.54) | | 0.02 | <0.001 | 1.10 (1.07, 1.13) | 0.02 | <0.001 | |
|  |  |  |  |  |  |  |  | |  |  |  |  |  | |
| **History of psychotic disorder** | 0.94 (0.91, 0.96) | 0.01 | <0.001 | 2.70 (2.58, 2.83) | 0.06 | <0.001 | 0.98 (0.96, 1.00) | | 0.01 | 0.039 | 0.86 (0.84, 0.88) | 0.01 | <0.001 | |
|  |  |  |  |  |  |  |  | |  |  |  |  |  | |
| **History of anxiety disorder** | 1.09 (1.06, 1.12) | 0.01 | <0.001 | 1.27 (1.21, 1.32) | 0.03 | <0.001 | 0.87 (0.85, 0.89) | | 0.01 | <0.001 | 1.28 (1.24, 1.31) | 0.02 | <0.001 | |
|  |  |  |  |  |  |  |  | |  |  |  |  |  | |
| **History of delirium** | 0.84 (0.82, 0.86) | 0.01 | <0.001 | 1.37 (1.32, 1.43) | 0.03 | <0.001 | 1.18 (1.16, 1.20) | | 0.01 | <0.001 | 0.89 (0.87, 0.92) | 0.01 | <0.001 | |
|  |  |  |  |  |  |  |  | |  |  |  |  |  | |
| **History of behavioural problems** | 1.24 (1.19, 1.29) | 0.02 | <0.001 | 1.11 (1.05, 1.18) | 0.03 | <0.001 | 1.03 (1.00, 1.06) | | 0.01 | 0.029 | 1.02 (0.98, 1.05) | 0.02 | 0.344 | |
|  |  |  |  |  |  |  |  | |  |  |  |  |  | |
| **History of personality disorders** | 1.02 (0.99, 1.05) | 0.01 | 0.119 | 1.13 (1.09, 1.17) | 0.02 | <0.001 | 1.29 (1.27, 1.31) | | 0.01 | <0.001 | 1.09 (1.06, 1.11) | 0.01 | <0.001 | |
| * Wald test for variable inclusion | | | | | | | | | | | | | | |
| ^✝^ “Unknown” category excluded from output | | | | | | | |  | | | | | |  |

# 4 Time to referral to residential aged care

Figure S2 shows the distribution of time until referral to residential aged care in our dataset (as determined by a mode_of_separation_recode value of 3 in the APDC, indicating referral to aged care), relative to the initial dementia diagnosis in hospital (for the dementia cohort) or self-harm diagnosis (for the self-harm cohort), split by those who enter go onto enter the dementia and self-harm subgroup, and those that do not. For those that entered the dementia and self-harm subgroup, we also plot the time to referral to residential aged care relative to their second diagnosis (i.e., self-harm for those in the dementia cohort and dementia for those in the self-harm cohort).

On visual inspection, it appears there is a peak of referrals to residential aged care for those that enter the dementia and self-harm subgroup when receiving the second diagnosis (particularly for those in the self-harm cohort) that is not evident for this subgroup at the time of their initial diagnosis (i.e., dementia for the dementia cohort or self-harm for the self-harm cohort). This examination of the data is entirely cursory and exploratory, and is presented only to probe one of many possible reasons why hospital service use may change following the second diagnosis. No strong inference should be drawn from figure S2.

## Figure S2: Density of time to referral to residential aged care relative to time of first and second diagnosis for both the dementia cohort and self-harm cohort.
